# Bioactivity-Guided Isolation of Stigmasterol from *Bursera bipinnata* Resin: Pharmacological Evidence for Wound-Healing Activity

**DOI:** 10.3390/ph19060931

**Published:** 2026-06-12

**Authors:** Luis Rubén Martínez-Cuevas, María Crystal Columba-Palomares, Baldomero Esquivel-Rodríguez, Alejandro Pérez-Feria, Vera L. Petricevich, Edda Sciutto, José Alejandro Espinosa-Cerón, Verónica Rodríguez-López

**Affiliations:** 1 Laboratorio de Farmacognosia y Química de Productos Naturales, Facultad de Farmacia, Universidad Autónoma del Estado de Morelos, Cuernavaca 62209, Morelos, Mexico; luis.ruben123b@gmail.com (L.R.M.-C.); cpmc_ff@uaem.mx (M.C.C.-P.); 2 Departamento de Productos Naturales, Instituto de Química, Universidad Nacional Autónoma de México, Circuito Exterior, Ciudad Universitaria, Ciudad de Mexico 04510, Mexico; besquivel@iquimica.unam.mx; 3 Centro de Investigación en Biotecnología, Universidad Autónoma del Estado de Morelos, Cuernavaca 62210, Morelos, Mexico; ale970604@gmail.com; 4 Laboratorio de Inflamación y Toxicología, Facultad de Medicina, Universidad Autónoma del Estado de Morelos, Cuernavaca 62350, Morelos, Mexico; vera.petricevich@uaem.mx; 5 Departamento de Inmunología, Instituto de Investigaciones Biomédicas, Universidad Nacional Autónoma de México, Ciudad de Mexico 04510, Mexico; edda@unam.mx (E.S.); alexec2803@gmail.com (J.A.E.-C.)

**Keywords:** *Bursera bipinnata*, anti-inflammatory, stigmasterol, lupane, triterpenoids, wound-healing, terpenoids, antibacterial, bioactive compounds

## Abstract

**Background/Objectives**: *Bursera bipinnata* (DC.) Engl. resin (locally known as “copal blanco”) is traditionally used in Mexican ethnomedicine to treat infected wounds and skin inflammation, but the bioactive constituents underlying these effects remain largely uncharacterized. This study aimed to identify the compounds responsible for the wound-healing properties of the resin through bioactivity-guided fractionation and to evaluate their anti-inflammatory and antibacterial activities as complementary mechanisms supporting tissue repair. **Methods**: Crude resin (1.2–5.0 mg/mL) was assayed for anti-inflammatory activity in the TPA-induced ear-edema model in BALB/c mice, for antibacterial activity (MIC) against six clinically relevant strains, and for wound-healing activity in a murine excisional model with pirfenidone (PFD) as the reference drug (*n* = 5 per group). Bioactivity-guided fractionation followed by spectroscopic elucidation (^1^H- and ^13^C-NMR, IR, EI-MS) led to the isolation of five constituents. Stigmasterol, the most active compound, was subsequently evaluated in an LPS-induced systemic inflammation model (oral administration, 20 mg/kg/day × 3 days) to characterize its immunomodulatory profile (TNF-*α*, IL-1*β*, IL-6, IFN-*γ*, IL-10) and in the wound-healing model to quantify local IL-6, IL-10 and TGF-*β*1 in skin homogenates. **Results**: The crude resin (5.0 mg/mL) achieved 99.63% wound closure at day 12 and a 49.08% reduction in TPA-induced ear edema, comparable to indomethacin (55.76%). The resin displayed selective antibacterial activity against *Streptococcus pyogenes* (MIC 125 µg/mL) and *Salmonella typhimurium* (MIC 250 µg/mL). Bioactivity-guided fractionation yielded the phytosterol stigmasterol (**1**), three lupane-type triterpenoids (lupeol acetate (**2**), lupenone (**3**), 3-epilupeol (**5**)), and the sesquiterpenoid caryophyllene oxide (**4**). At an equimolar 1 µM concentration, stigmasterol (**1**) shortened the mean wound-healing time to 10.3 ± 0.4 days, comparable to pirfenidone, and was associated with attenuation of systemic TNF-*α*, IL-1*β* and IL-6 peaks and with sustained local IL-10 and TGF-*β*1 expression. Histological assessment confirmed accelerated re-epithelialization and improved collagen organization. The resin was non-irritant in the OECD 404 acute dermal test (Primary Irritation Index = 0.00). **Conclusions**: These findings provide pharmacological evidence supporting the traditional use of *B. bipinnata* resin for wound healing. Stigmasterol (**1**), together with the lupane-type triterpenoids lupenone (**3**) and 3-epilupeol (**5**), were identified as key bioactive constituents. The data are consistent with a coordinated immunomodulation, in which stigmasterol is associated with reduced systemic pro-inflammatory signalling and increased local IL-10/TGF-*β*1 expression, an interpretation that should be confirmed in chronic and impaired wound-healing models.

## 1. Introduction

Mexico is home to approximately 33,110 plant species, of which roughly 87 belong to the *Bursera* genus [1,2]. Among them, *Bursera bipinnata*, commonly known as “copal blanco,” plays a prominent role in Mesoamerican traditional medicine and ritual practices. Historically, the resin has been burned as incense for purification and used in traditional Mexican medicine to treat wounds, oral hemorrhages, and infections, owing to its reported antiseptic and anti-inflammatory properties [2,3,4]. Beyond its cultural relevance, the resin is an important non-timber forest product for rural communities [5].

Evidence from phytochemical studies indicates that the *Bursera* genus is a rich source of bioactive terpenoids. Compounds such as *β*-caryophyllene, *α*-amyrin, and lupeol have been reported to promote tissue repair through anti-inflammatory and wound-healing effects, predominantly documented in vitro models and pointing to a multi-target mechanism of action that requires validation in in vivo systems [6,7]. Cutaneous wound healing is a complex process involving coordinated stages of hemostasis, inflammation, proliferation, and remodeling [8]. Although medicinal plants rich in terpenoids have shown the potential to enhance these processes [9], the specific chemical identity of the healing agents within *B. bipinnata* resin and the precise molecular pathways they influence have not been established. Specifically, it remains unknown how the complex mixture of terpenoids and phytosterols in this species coordinates the transition from inflammation to tissue repair.

A key challenge in wound care is the management of chronic wounds, which are often characterized by a persistent inflammatory state. In this context, triterpenoids such as *α*-amyrin have been reported to exert significant anti-inflammatory effects by modulating the production of pro-inflammatory cytokines and reducing oxidative stress [10]; however, their specific role in orchestrating inflammatory mediators during the wound-healing timeline remains poorly understood. Elucidating these interactions is essential to support the ethnopharmacological use of “copal blanco” and to identify bioactive leads capable of modulating the inflammatory microenvironment in cutaneous wounds.

The pharmacological potential of *Bursera* resins is traditionally linked to their complex chemical profile, characterized by a diverse array of mono-, sesqui-, di- and triterpenoids. In related species, these secondary metabolites, specifically terpenoids and sterols, have shown a remarkable capacity to modulate the inflammatory microenvironment, facilitating the transition from tissue injury to repair [11,12]. Given this background, a bioactivity-guided fractionation approach was selected as a rational strategy to identify the specific bioactive constituents responsible for the resin’s therapeutic effects. This systematic process allows for the isolation of lead compounds that can be evaluated not only for their intrinsic potency but also for their specific roles in immunological homeostasis.

The present study evaluates the wound-healing and anti-inflammatory potential of *B. bipinnata* resin through a bioactivity-guided fractionation approach. It was hypothesized that the medium-polarity fraction of *B. bipinnata* resin contains compounds that accelerate cutaneous wound closure by attenuating early pro-inflammatory cytokines and sustaining local TGF-*β*1 expression. Furthermore, the study explores antibacterial activity as a complementary defensive mechanism. This research aims to provide pharmacological evidence supporting the traditional use of “copal blanco” in wound management.

## 2. Results

### 2.1. Chemical Profiling of the Resin and Bio-Guided Fractionation

#### 2.1.1. Chemical Composition of *B. bipinnata* Resin (GC-MS Analysis)

The initial characterization of *B. bipinnata* resin was performed through Gas Chromatography coupled with Mass Spectrometry (GC-MS), resulting in the tentative assignment of constituents based on mass spectral similarity with the NIST library database.

Among the monoterpenoids, D-limonene (19.15%) was the major constituent, a compound well-documented for its antioxidant and anti-inflammatory effects [13,14]. Additionally, the presence of *β*-caryophyllene (9.61%) and caryophyllene oxide (8.23%) were detected; the former acts as a selective CB_2_ receptor agonist [15,16], while the latter is recognized for its antimicrobial efficacy [17].

Within the triterpenic fraction, a significant peak (19.60%) was preliminarily assigned as lupeol. However, because the chromatographic conditions and spectral similarity with the NIST database do not allow for the resolution of certain epimers, this signal likely represents an unresolved pair of lupeol and 3-epilupeol. This underscores the limitation of GC-MS for the unequivocal identification of such isomers, which are not distinguishable by this technique alone. Lupeol-type triterpenoids are extensively described as modulators of the inflammatory response [18]. Furthermore, lupeol acetate (18.00%) has been linked to both anti-inflammatory and antibacterial activities [19].

Given that the diverse chemical ensemble within the resin suggests a multi-target synergy, but also contains constituents with potential structural ambiguity, a bioactivity-guided approach was implemented. This strategy was essential to determine whether specific isolated molecules, now definitively identified through spectroscopic methods, independently modulate key pathways of the healing process.

#### 2.1.2. Bioactivity-Guided Fractionation of *Bursera bipinnata* Resin: Isolation of Bioactive Compounds with Wound Healing Potential

The pharmacological assessment of the wound-healing potential began with the evaluation of the crude *B. bipinnata* resin. For this purpose, three scaled doses: 1.2, 2.5, and 5.0 mg/mL were implemented. The selection of these dosages followed an escalation strategy designed to determine a potential dose-dependent relationship and to identify the optimal pharmacological concentration for promoting tissue repair.

**Figure 1:**
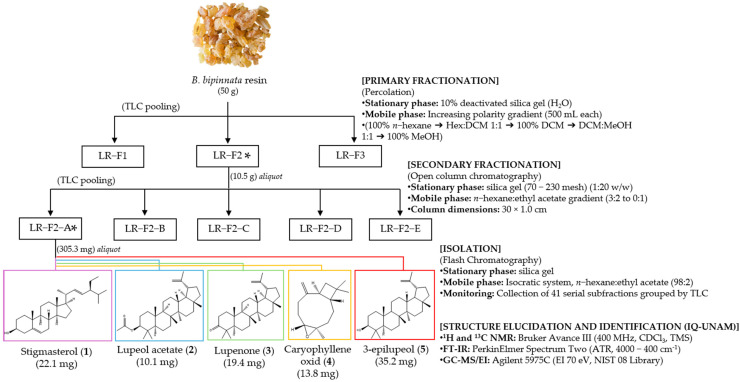
Schematic representation of the bioactivity-guided isolation of bioactive terpenoids and phytosterols from *B. bipinnata* resin. * Indicates the most effective fraction and subfractions identified during the bioactivity-guided fractionation.

Among the tested treatments, the 5.0 mg/mL concentration exhibited the most prominent regenerative activity in the in vivo models. Consequently, this specific concentration was selected as the starting point for the subsequent bioactivity-guided fractionation (Figure 1). This systematic approach ensured that the identification of active constituents was directly linked to the demonstrated efficacy of the whole resin, thereby bridging the gap between traditional use and molecular characterization.

The crude resin (50.0 g) was initially fractionated through a sequential extraction process using solvents of increasing polarity, yielding three primary fractions: LR-F1 (26.45 g, 52.9% *w*/*w*), LR-F2 (18.12 g, 36.2% *w*/*w*), and LR-F3 (4.23 g, 8.5% *w*/*w*). The resulting fractions were monitored and grouped according to their chemical profiles via Thin Layer Chromatography (TLC). Among these, LR-F2, corresponding to the medium-polarity group obtained predominantly with dichloromethane, exhibited the most prominent biological activity in the in vivo wound-healing model.

As shown in Figure 1, following the identification of LR-F2 as the most active primary fraction, an aliquot of 10.5 g was subjected to a second stage of purification via Open Column Chromatography (OCC) using silica gel as the stationary phase and a gradient elution system. This process yielded five subfractions: LR-F2-A (1.90 g, 6.55% *w*/*w*), LR-F2-B (1.17 g, 4.03% *w*/*w*), LR-F2-C (2.69 g, 9.11% *w*/*w*), LR-F2-D (1.79 g, 6.17% *w*/*w*), and LR-F2-E (2.49 g, 8.59% *w*/*w*). Among these, subfraction LR-F2-A exhibited the most significant pharmacological activity, achieving 95.44% wound closure by day 12. Notably, subfraction LR-F2-E also demonstrated substantial healing potential, reaching 93.05% closure at the same time point. While the high efficacy of LR-F2-E suggests it is a promising candidate for future phytochemical exploration, LR-F2-A was prioritized for subsequent isolation and structural elucidation due to its superior performance in the in vivo model.

**Figure 2:**
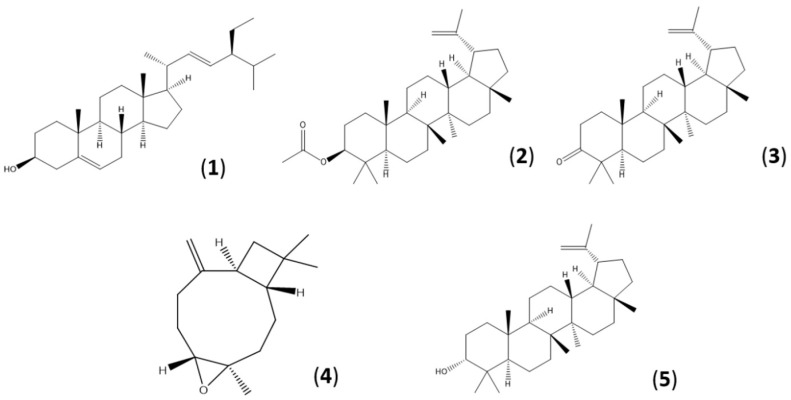
Chemical structures of the constituents isolated from the LR-F2-A subfraction of *B. bipinnata* resin: the phytosterol stigmasterol (**1**); the triterpenoids lupeol acetate (**2**), lupenone (**3**), and 3-epilupeol (**5**); and the sesquiterpenoid caryophyllene oxide (**4**).

Furthermore, although this study focused on the primary markers of the bioactive group, the identification of minor compounds within LR-F2-A remains an open area of interest to fully characterize the synergistic potential of its chemical matrix. Consequently, to identify the specific molecules responsible for the observed repair process, an aliquot of the bioactive subfraction LR-F2-A (305.3 mg) was subjected to isocratic Flash Column Chromatography (FCC). This targeted strategy facilitated the successful isolation of five main constituents, with their respective absolute yields and percentages relative to the initial crude resin: stigmasterol (**1**, 22.1 mg, 0.47% *w*/*w*; 95% purity), lupeol acetate (**2**, 10.1 mg, 0.21% *w*/*w*; 92% purity), lupenone (**3**, 19.4 mg, 0.40% *w*/*w*; 97% purity), caryophyllene oxide (**4**, 13.8 mg, 0.27% *w*/*w*; 96% purity), and 3-epilupeol (**5**, 35.2 mg, 0.75% *w*/*w*; 95% purity) (Figure 2). This range is considered consistent with the technical limitations associated with the absolute separation of structural isomers commonly found in *Bursera* resins.

Structural elucidation was confirmed through advanced characterization techniques, including Infrared Spectroscopy (IR), Proton and Carbon-13 Nuclear Magnetic Resonance (^1^H and ^13^C NMR), and Electron Ionization Mass Spectrometry (EIMS). Detailed spectroscopic properties and complete spectra are provided in the Supplementary Materials (Tables S2–S6 and Figures S2–S21).

Due to the limited mass obtained for lupeol acetate (**2**) and caryophyllene oxide (**4**), these compounds were utilized exclusively for structural elucidation and chemical verification. Conversely, stigmasterol (**1**), lupenone (**3**), and 3-epilupeol (**5**) were prioritized for in vivo pharmacological evaluation. This selection ensured sufficient material for a standardized topical application protocol, which was administered during the critical early phase of healing (days 1–3 post-wounding, *n* = *5*).

Following this initial treatment phase, all experimental groups underwent longitudinal monitoring until day 14 to capture the complete progression toward wound closure and determine the Mean Wound Healing Time (MWHT). In the murine model, these compounds exhibited significant wound-healing potential. Notably, stigmasterol (**1**) was associated with a pronounced acceleration of wound closure from day five onwards, comparing favorably to the pirfenidone (PFD) positive control.

This bioactivity-guided approach proved essential for the selective isolation of constituents with specific biological activity. By correlating molecular structures with pharmacological effects, this targeted strategy facilitates the identification of key bioactive drivers within the complex resin matrix. Furthermore, this methodology accounts for environmental variables, such as the specific collection site in Morelos, Mexico, which is reported to influence the concentration and efficacy of markers like stigmasterol (**1**) and lupenone (**3**) in *B. bipinnata* [20]. Consequently, the isolation of specific compounds ensures the chemical reproducibility and potency required for characterizing natural product-based therapies. While the full therapeutic potential of the resin could be further explored, acknowledging that the complex matrix likely contains additional bioactive metabolites, the constituents identified here demonstrate substantial relevance and a significant impact on the wound-healing process.

### 2.2. Determination of the Anti-Inflammatory Activity of Bursera bipinnata Resin in a TPA-Induced Mouse Ear Edema Model

The anti-inflammatory activity of *B. bipinnata* resin was evaluated using the 12-*O*-tetradecanoylphorbol-13acetate (TPA)-induced mouse ear edema model at doses of 0.03, 0.10, and 0.30 mg/ear. Indomethacin (0.10 mg/ear) served as the positive control (Table 1). The results revealed that the resin exhibited dose-dependent anti-inflammatory activity. The highest dose (0.30 mg/ear) achieved 49.08%, compared to 55.76% observed for indomethacin.

### 2.3. Antibacterial Activity of Bursera bipinnata Resin

The antibacterial potential of *B. bipinnata* resin was evaluated against six clinically relevant pathogenic strains, including three Gram-positive (*Staphylococcus aureus* ATCC 6538, methicillin-resistant *S. aureus* MRSA ATCC 43300, and *Streptococcus pyogenes* ATCC 19615) and three Gram-negative bacteria (*Escherichia coli* ATCC 8739, *Pseudomonas aeruginosa*, and *Salmonella typhimurium* ATCC 14028). These strains were selected due to their high prevalence in skin and wound infections, where pathogens such as MRSA and *P. aeruginosa* often complicate the healing process through multidrug resistance [21,22,23,24].

As summarized in Table 2, the resin exhibited selective but moderate antibacterial activity. The Minimum Inhibitory Concentration (MIC) values were 125 µg/mL for *S. pyogenes* and 250 µg/mL for *S. typhimurium*. Although these inhibitory concentrations represent a moderate effect when compared to the high potency of the reference antibiotic gentamicin (MIC 0.32 µg/mL), they suggest a relevant supplementary benefit [25,26].

### 2.4. Evaluation of the Wound-Healing Activity of Bursera bipinnata Resin in a Murine Excisional Wound Model

The relative scarcity of scientific literature specifically addressing the wound-healing properties of isolated constituents from *B. bipinnata* provided a compelling rationale for a bioactivity-guided investigation. Consequently, the crude resin was subjected to a systematic evaluation to identify the primary chemical drivers of tissue repair. For this evaluation, animals were assigned to treatment groups through a randomization process using a random number table, ensuring an unbiased distribution across all experimental conditions. The wound-healing activity of *B. bipinnata* resin was evaluated at doses of 1.2, 2.5, and 5.0 mg/mL in a model recognized for its reliability in simulating in vivo dynamics [27,28]. Pirfenidone (PFD) at 0.08 g/mL and an untreated group served as positive and negative controls, respectively. Each treatment (20 µL) was topically applied once daily for the first three days post-induction to explore potential dose-dependent effects [29,30,31]. As presented in Table 3, all resin doses significantly increased wound contraction compared to the negative control (* *p* < 0.05). The 5.0 mg/mL dose achieved 77.24% closure by day 5, compared to 72.67% in the PFD group. By day 12, the 5.0 mg/mL dose reached 99.63% wound closure. On day 2, the 5.0 mg/mL dose showed a contraction of 36.55%, which was higher than the 14.76% and 20.38% observed for the 1.2 mg/mL and 2.5 mg/mL doses, respectively. Quantitatively, PFD showed higher contraction percentages during days 2–3, while the resin treatments exhibited more pronounced contraction rates from day 7 through day 12.

**Figure 3:**
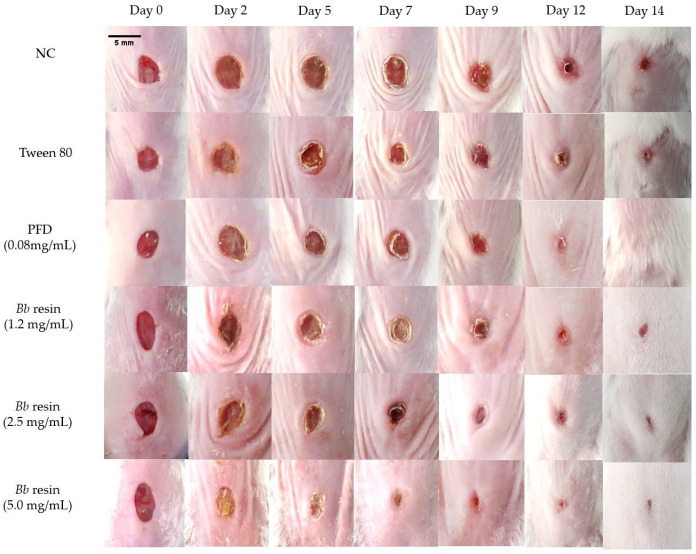
Macroscopic evaluation of wound healing progression following topical treatments with *B. bipinnata* resin. Representative images illustrating the healing process recorded on days 0, 2, 5, 7, 9, 12, and 14 for the following groups: negative control (NC), vehicle (Tween 80), pirfenidone (PFD, 0.08 g/mL), and *B. bipinnata* resin (1.2, 2.5, and 5.0 mg/mL). Scale bar = 5 mm. The initial wound diameter served as the internal reference for planimetric analysis.

Figure 3 shows the wound healing process in mice, with photographs of each treatment from initial wound induction (day 0) to the conclusion of the experiment (day 14). By day two, all groups, except those treated with Tween 80 and 2.5 mg/mL resin, exhibited some degree of oozing in at least one mouse, likely due to animal movement and interactions. Edema and redness persisted throughout the experiment in at least one mouse from negative control, Tween 80, 1.2 mg/mL resin, and 2.5 mg/mL resin groups. No bleeding was observed in any group. Scab formation began earlier in the PFD, 5.0 mg/mL resin, and 2.5 mg/mL resin groups, initiating on day five, suggesting more rapid activation of tissue repair mechanisms. In contrast, scab formation occurred progressively over time in the remaining groups. Table S8 in the Supplementary Materials provides a detailed analysis of these findings by further evaluating the clinical parameters of wound healing. These results suggested that the resin possesses significant regenerative potential, particularly at the highest concentration tested, which served as the basis for the subsequent fractionation and isolation of bioactive markers.

### 2.5. Evaluation of the Wound-Healing Activity of Primary Fractions of Bursera bipinnata Resin in a Murine Model

The wound-healing activity of the three primary fractions (LR-F1, LR-F2, and LR-F3) obtained from *B. bipinnata* resin was evaluated to identify the bioactive constituents responsible for the therapeutic effects of the crude material. To ensure an unbiased distribution of the experimental units, mice were assigned to treatment groups through a randomization process using a random number table. Each fraction was incorporated into a Tween 80 vehicle at 5.0 mg/mL and applied topically (20 µL) during the first three days post-induction. These treatments were compared against a negative control group and a positive control (pirfenidone, PFD, 0.08 g/mL).

As summarized in Table 4, all fractions demonstrated significantly enhanced wound healing compared to the negative control (* *p* < 0.05), as determined by one-way ANOVA followed by Dunnett’s post hoc test. Among the series, LR-F2 exhibited the highest degree of wound contraction (30.05%) between days two and nine, representing a substantial acceleration in the repair process compared to the untreated group. While LR-F1 (11.56%) and LR-F3 (14.04%) also showed significant activity, LR-F2 proved to be the most effective driver of early tissue contraction. By day twelve, the contraction percentages for LR-F1 (98.93%), LR-F2 (96.84%), and LR-F3 (96.12%) were not only significantly higher than those of the negative control, but also comparable to the values observed for the PFD group (99.83%). These results confirm that the fractionation process successfully concentrated bioactive molecules capable of driving efficient tissue regeneration far exceeding the natural repair rate of the untreated group.

**Figure 4:**
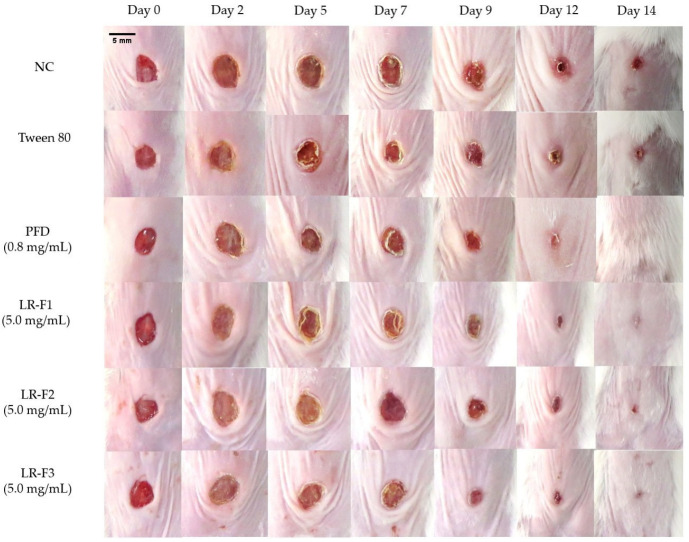
Macroscopic evaluation of wound healing in mice treated topically with *B. bipinnata* resin fractions. Representative images showing the wound healing process recorded on days 0, 2, 5, 7, 9, 12, and 14 for the following groups: negative control (NC), vehicle (Tween 80), pirfenidone (PFD, 0.08 g/mL), and *B. bipinnata* resin fractions LR-F1, LR-F2, and LR-F3 (5.0 mg/mL each). Scale bar = 5 mm. The initial wound diameter served as the internal reference for planimetric analysis.

The progression of wound healing is depicted in Figure 4. No clinical signs of infection or exudate were observed in any treatment group. While the negative control group exhibited delayed healing and failed to reach complete closure within the study period, all resin-treated groups achieved closure levels comparable to the positive control by day twelve. Specific clinical observations included transient edema in the LR-F1 and LR-F2 groups, with swelling in the former persisting until day seven. Scab formation was evident by day two, with LR-F2 demonstrating the most rapid early-stage healing among the tested fractions. Detailed clinical parameters and daily observational data for each experimental group are summarized in Table S9 of the Supplementary Materials.

Given the superior pharmacological performance of the medium-polarity fraction (LR-F2) during the critical early phases of the healing process, it was determined necessary to further refine its chemical composition. This prioritized subfractionation was implemented to isolate and identify the specific chemical markers within LR-F2 that contribute most significantly to its regenerative properties, facilitating a more detailed understanding of the individual molecular drivers involved in tissue repair.

### 2.6. Evaluation of the Wound Healing Activity of Subfractions Derived from the LR-F2 Fraction of Bursera bipinnata Resin

The wound-healing potential of the subfractions derived from the LR-F2 fraction (LR-F2-A to LR-F2-E) was evaluated after they were grouped by chemical affinity via TLC. To ensure the objectivity of the study, animals were assigned to the experimental groups through a randomization process using a random number table. These subfractions were topically applied at a concentration of 5.0 mg/mL (20 µL) for the first three days following wound induction, and their efficacy was evaluated primarily against a negative control group, with pirfenidone (PFD) serving as a reference.

As summarized in Table 5, most subfractions demonstrated a significant acceleration in wound contraction compared to the negative control (* *p* < 0.05). Specifically, LR-F2-A and LR-F2-E exhibited the most pronounced effects, with contraction rates of 29.71% and 18.72%, respectively, by day two. These values represent a significant increase in activity during the initial inflammatory phase compared to the untreated group. In contrast, LR-F2-B and LR-F2-C presented moderate contraction rates (11.66% and 11.92%), whereas LR-F2-D demonstrated the weakest response (2.49%), showing no significant difference relative to the negative control in the early stages. Both LR-F2-A and LR-F2-E continued to promote tissue repair throughout the proliferative and remodeling phases, achieving closure rates of 95.44% and 93.05%, respectively, by day twelve.

Among the series, LR-F2-A exhibited the highest overall efficacy, with a Mean Wound-Healing Time (MWHT) of 11.4 ± 0.5 days, which was significantly shorter than the time required for the negative control group and comparable to the 10.5 ± 0.4 days observed for the reference drug pirfenidone. While the high efficacy of LR-F2-E suggests it is a promising candidate for future phytochemical exploration, LR-F2-A was prioritized for subsequent isolation and structural elucidation due to its superior performance in this in vivo model. Furthermore, although this study focused on the primary chemical markers within this bioactive group, the identification of minor compounds within LR-F2-A remains a relevant area for future investigation to fully characterize the synergistic potential of its chemical matrix.

**Figure 5:**
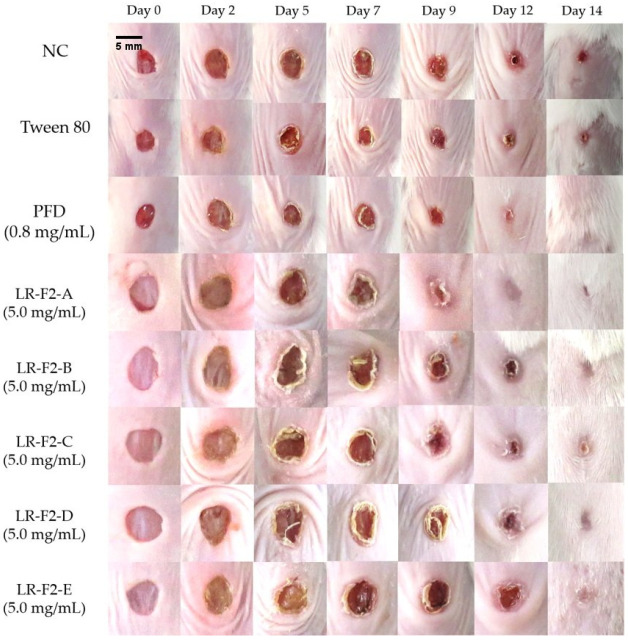
Macroscopic evaluation of wound healing in mice treated with subfractions of LR-F2 resin. Representative images illustrating the healing process recorded on days 0, 2, 5, 7, 9, 12, and 14 for the following groups: negative control (NC), vehicle (Tween 80), pirfenidone (PFD, 0.08 g/mL), and LR-F2 subfractions (5.0 mg/mL each). Scale bar = 5 mm. The initial wound diameter served as the internal reference for planimetric analysis.

The macroscopic progression of wound healing is illustrated in Figure 5. No signs of infection were observed in any treatment group from day zero to day fourteen. While exudate formation was noted in the negative control and Tween 80 groups during the initial days, edema and redness were primarily limited to the early stages of healing (days 2–7) across the first three subfractions and control groups, gradually diminishing as repair progressed. Scab formation was initiated as early as day two in the PFD, LR-F2-A, and LR-F2-E groups, suggesting a more rapid activation of tissue repair mechanisms, whereas scab formation in the remaining groups was delayed until day seven. Further details on the clinical parameters assessed throughout the study are provided in Supplementary Table S10.

The superior performance of subfraction LR-F2-A, particularly its ability to promote significant early wound contraction and complete closure within a timeframe comparable to the positive control, justified its selection for further chemical characterization. Consequently, a detailed isolation process was implemented to identify the specific secondary metabolites within this subfraction and determine their individual pharmacological contributions to the regenerative process.

### 2.7. Evaluation of the Wound Healing Activity of Compounds Isolated from Bursera bipinnata Resin

Among the five compounds characterized in the bioactive subfraction LR-F2-A (stigmasterol **1**, lupeol acetate **2**, lupenone **3**, caryophyllene oxide **4** and 3-epilupeol **5**), compounds **1**, **3** and **5** were prioritized for in vivo evaluation on the basis of their higher chromatographic yields, which allowed for a standardized application throughout the 14-day excisional protocol. The remaining two constituents (**2** and **4**) were not advanced to the in vivo phase owing to insufficient isolated mass (Section 3.4, Limitation 7). To compare the intrinsic pharmacological potency of the three selected isolates on an equimolar basis, a topical concentration of 1 µM was used for all treatments.

Animals were assigned to treatment groups using a random number table, and the same negative control (NC) from previous stages were utilized to minimize the total number of experimental units, adhering to ethical guidelines for animal reduction. Quantitative wound contraction data and the Mean Wound Healing Time (MWHT) are summarized in Table 6. Stigmasterol (**1**) exhibited the highest efficacy, achieving 66.44% contraction by day five and 96.84% wound closure by day 12, with a MWHT of 10.3 ± 0.4 days. Compounds **5** and **3** also significantly accelerated contraction compared to the negative control (NC), reaching 95.74% (MWHT 10.6 ± 0.5 days) and 92.16% (MWHT 10.9 ± 0.6 days) closure, respectively.

It is worth noting that, when tested at the same equimolar concentration (1 µM), all three isolated compounds (**1**, **3**, and **5)** demonstrated wound-healing activity. Stigmasterol (**1**) showed the best performance, followed by 3-epilupeol (**5**), and finally lupenone (**3**). The equimolar comparison at 1 µM, although methodologically rigorous [32], should be interpreted with certain caveats: (i) 1 µM is below the EC_50_ reported for pirfenidone in wound healing assays (≈10–100 µM; [33], so we do not make a comparison against PFD; (ii) differences in molecular size, lipophilicity (logP) and skin permeability between stigmasterol, lupane-type triterpenoids and pirfenidone may result in non-equivalent tissue exposures even at identical applied molar concentrations; (iii) A complete dose–response curve (0,1, 1, 10, and 50 µM) is required for each compound to calculate ED_50_ values and allow for a fully validated potency classification. These caveats are explicitly identified as priorities for future work.

**Figure 6:**
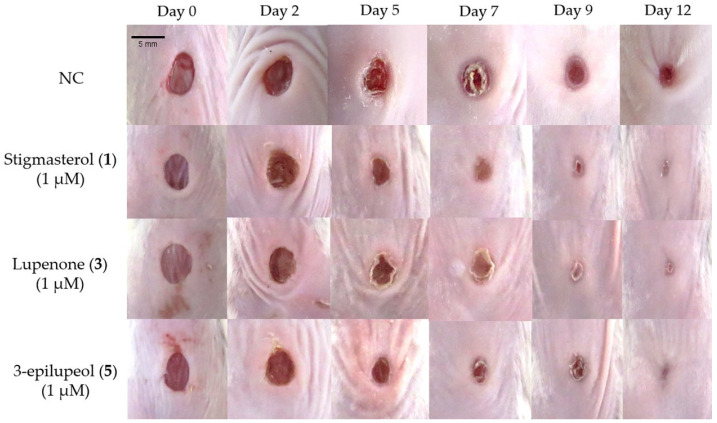
Macroscopic evaluation of wound healing in mice treated with compounds isolated from the most active subfraction of *B. bipinnata* resin (LR-F2-A). Representative images illustrating the healing process recorded on days 0, 2, 5, 7, 9, 12, and 14 for the following groups: negative control (NC), vehicle (Tween 80), pirfenidone (PFD, 1 μM), and compounds stigmasterol (**1**), lupenone (**3**), and 3-epilupeol (**5**) (1 μM each). Scale bar = 5 mm. The initial wound diameter served as the internal reference for planimetric analysis.

**Figure 7:**
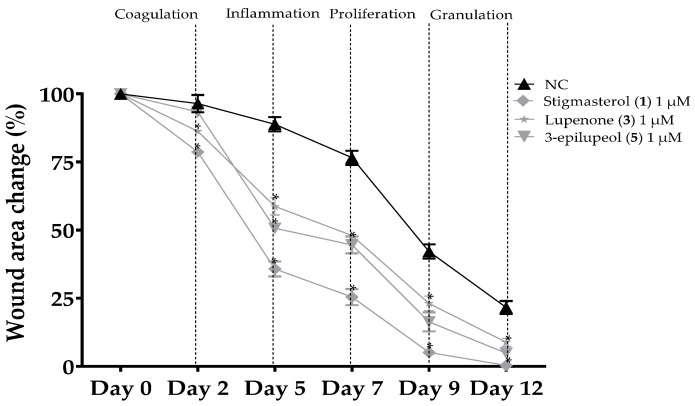
Healing kinetics were determined on the basis of the percentage of wound closure treated with the compounds isolated from *B. bipinnata* resin, stigmasterol (**1**), lupenone (**3**), and 3-epilupeol (**5**), compared with the negative control (NC) in a murine model. The data are shown with the S.E.M. (*n* = 5) and * *p* < 0.05 indicates a significant difference relative to the NC; one-way ANOVA followed by Dunnett’s post hoc test was used.

Macroscopic monitoring (Figure 6) revealed progressive contraction across all treatment groups, with no apparent signs of hemorrhage or infection. Scab formation occurred between days two and seven in all treated animals, indicating a standard progression through the inflammatory and proliferative phases. A detailed qualitative evaluation is presented in Supplementary Table S11. As illustrated in the wound closure kinetics (Figure 7), stigmasterol (**1**) was associated with a more pronounced acceleration of the healing process compared to the other compounds. This consistent downward trend suggests a more rapid resolution of the inflammatory phase. While mild edema was sporadically recorded in the NC group and in one mouse treated with **5**, these observations did not interfere with the overall progression toward closure.

To the best of current knowledge, specialized scientific literature has not extensively explored the individual bioactive constituents of *B. bipinnata* resin, despite its long-standing history in traditional medicine. While it is plausible that other metabolites within the complex resin matrix including those not isolated in the present study may possess specialized biological activity, the results obtained here underscore the pharmacological relevance of the isolated compounds. Specifically, the consistent regenerative activity demonstrated by stigmasterol (**1**) suggests that this phytosterol could be among the constituents contributing to the observed healing process. Consequently, it was selected for the subsequent biochemical evaluations to further explore the potential mechanisms of action involved in tissue repair.

### 2.8. Histological Assessment of Wound Repair Progression

**Figure 8:**
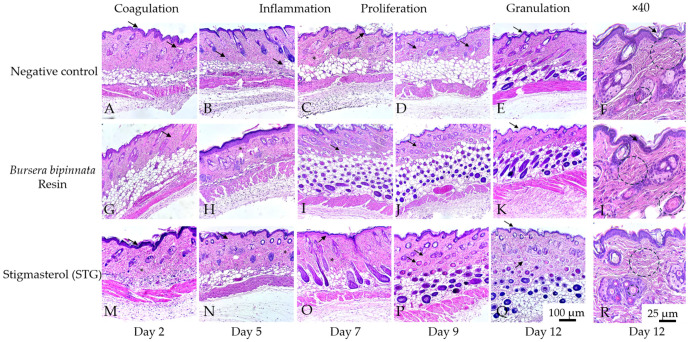
Representative H&E-stained sections illustrating the temporal progression of cutaneous wound healing across coagulation, inflammation, proliferation, granulation, and remodeling phases in control, *B. bipinnata* resin-treated, and stigmasterol-treated mice. Scale bars: 100 µm for 10× objective (Panels (**A**–**Q**)) and 25 µm for 40× objective (Panels (**F**,**L**,**R**)).

To further elucidate the microscopic mechanisms underlying the accelerated wound closure previously observed, a histological assessment was conducted using hematoxylin and eosin (H&E) staining. This analysis allowed for the monitoring of the classical phases of cutaneous wound healing in the murine model (Figure 8). The temporal progression of tissue repair revealed distinct morphological differences between the negative control (NC), the *B. bipinnata* resin group (5.0 mg/mL), and the stigmasterol (**1**) treated group.

During the early phase (Day 2), control tissues (Figure 8A) exhibited marked epidermal discontinuity and dense polymorphonuclear (PMN) infiltration. In contrast, the resin-treated group (Figure 8G) presented a thickened dermis rich in collagen fibers and evidence of early angiogenesis. Stigmasterol (**1**) treatment (Figure 8M) appeared to minimize initial structural degradation, showing a more compact dermal structure and a mild inflammatory infiltrate, suggesting a potential modulation of the acute response.

By Day 5, the inflammatory phase peaked in the control group (Figure 8B), characterized by pronounced interstitial edema. However, the treated groups demonstrated a significant reduction in edema (Table 7). The resin group (Figure 8H) promoted epithelial progression through hyperplasia in the stratum spinosum, while the stigmasterol (**1**) group (Figure 8N) exhibited a more efficient resolution of the inflammatory stage, with notable preservation of skin adnexa [34].

In the proliferative phase (Day 7), while the control group (Figure 8C) showed incomplete re-epithelialization, stigmasterol (**1**) (Figure 8O) was associated with the robust proliferation of anagen-phase hair follicles and an organized extracellular matrix (ECM). The resin group (Figure 8I) also showed advancements in dermal organization compared to NC.

By Day 9, differences in the maturity of the granulation tissue were evident; the resin group (Figure 8J) maintained active stromal remodeling with a persistent presence of inflammatory cells, indicating a sustained repair dynamic [35]. Meanwhile, the stigmasterol (**1**) group (Figure 8P) presented a dense ECM and a marked reduction in infiltration, surpassing the control in structural organization.

Finally, in the remodeling phase (Day 12), stigmasterol (**1**) treated tissues (Figure 8Q,R) achieved near-complete restoration of normal skin architecture, featuring mature sebaceous glands and organized collagen fibers, hallmarks of advanced healing [36]. The resin group (Figure 8K,L) similarly showed advanced closure, while the control group (Figure 8E,F) still exhibited signs of incomplete remodeling. These findings provide an integrated structural perspective on the therapeutic effects, further supported by the standardized histopathological scoring in Table 7.

### 2.9. Modulation of Local Cytokine Profiles (IL-6, IL-10, and TGF-β1) During Tissue Regeneration

**Figure 9:**
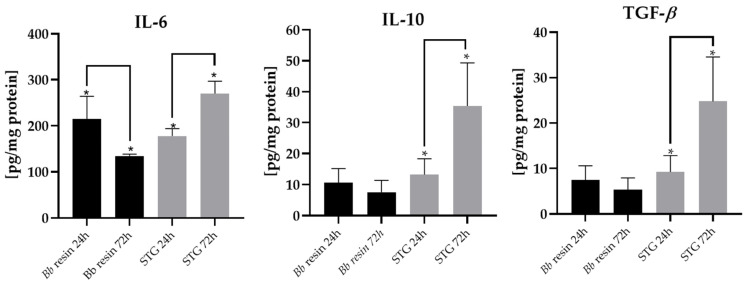
Comparative kinetics of local IL-6, IL-10, and TGF-*β*1 expression in wound tissue homogenates treated with *B. bipinnata* resin and stigmasterol (**1**). Cytokine concentrations (pg/mg protein) were assessed at 24 and 72 h post-injury. Values represent mean ± SEM (*n* = 5). Statistical significance was determined by one-way ANOVA (* *p* < 0.05).

To understand the molecular mechanisms underlying the accelerated tissue repair, the local expression of key inflammatory and proliferative markers was assessed at 24 and 72 h post-injury (Figure 9). The results revealed a distinct kinetic profile for stigmasterol (**1**) compared to the crude *B. bipinnata* resin, particularly during the transition from the inflammatory to the proliferative phase.

Interleukin-6 (IL-6) is a pleiotropic cytokine essential for initiating the wound-healing response and stimulating keratinocyte migration. At 24 h, both the resin and stigmasterol (**1**) groups showed comparable levels of IL-6. However, a significant divergence was observed at 72 h: while the resin-treated group exhibited a decline toward baseline, the stigmasterol (**1**) group showed a marked elevation (~270 pg/mg protein). This sustained IL-6 signaling at the 72 h mark is associated with the promotion of re-epithelialization and the early formation of the basement membrane, which aligns with the advanced epidermal thickness observed in the STG histological sections (Figure 8O) [37].

The resolution of the acute inflammatory response is partly regulated by IL-10, which limits tissue damage and promotes a regenerative environment. As illustrated in Figure 9, treatment with stigmasterol (**1**) induced a significant increase in local IL-10 concentrations by 72 h (~35 pg/mg protein), surpassing the levels observed in the resin-treated group. This upregulation suggests a more efficient transition toward the anti-inflammatory phase, consistent with the reduced edema and organized dermal structure previously identified in the STG-treated tissues [38].

The temporal profile of TGF-*β*1, a critical driver of extracellular matrix (ECM) synthesis, was similarly modulated. At 24 h, both treatments induced a comparable upregulation of this cytokine (* *p* < 0.05). However, by 72 h, a significant difference emerged: while TGF-*β*1 levels in the resin-treated group began to stabilize, the stigmasterol (**1**) group maintained a persistent elevation (~25 pg/mg protein). This sustained presence of TGF-*β*1 at the end of the initial treatment phase correlates with the robust collagen organization and the presence of mature hair follicles observed during the proliferative stage of repair.

### 2.10. Immunomodulatory Assessment of Stigmasterol (***1***) on the Pro- and Anti-Inflammatory Cytokine Profile in an LPS-Induced Systemic Inflammation Model

**Figure 10:**
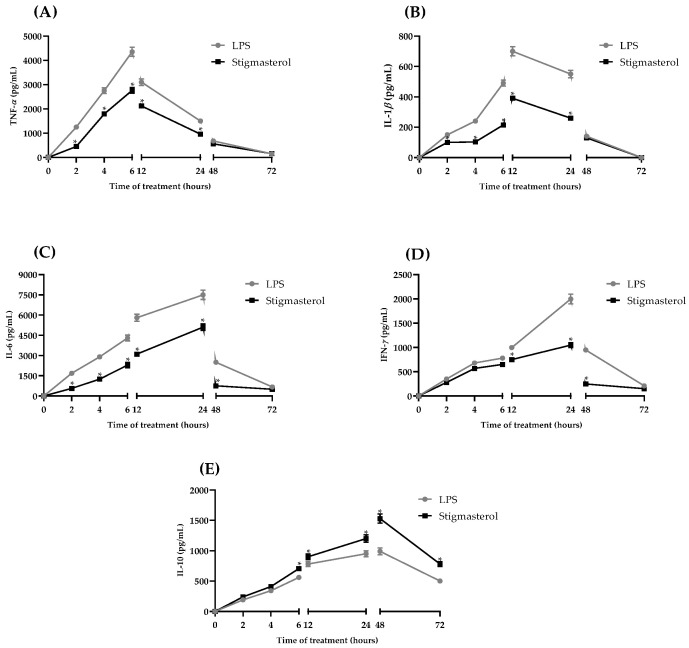
Modulatory effect of stigmasterol (**1**) on the kinetics of pro- and anti-inflammatory cytokine production in a murine model of systemic inflammation. (**A**) TNF-*α*, (**B**) IL-1*β*, (**C**) IL-6, (**D**) IFN-*γ* and (**E**) IL-10. Data are expressed as mean ± standard error of the mean (SEM). Statistical analysis was performed using two-way analysis of variance (ANOVA) to evaluate the effects of treatment and time, followed by Tukey’s post hoc test for multiple comparisons. Differences were considered statistically significant at (* *p* < 0.05).

To complement the findings observed in the localized wound-healing model and to further explore the intrinsic immunomodulatory capacity of stigmasterol (**1**), a complementary systemic pharmacology experiment was implemented. This study utilized a systemic inflammation challenge induced by lipopolysaccharide (LPS, 2.0 mg/kg) to evaluate the compound’s ability to modulate the inflammatory cascade independently of the mechanical aspects of tissue repair. While the previous experiments focused on topical application to assess localized effects, this systemic approach was selected to characterize the influence of orally administered stigmasterol (**1**) (20 mg/kg/day for three consecutive days before the LPS challenge) on the global cytokine response, utilizing a standardized LPS dose to ensure a robust and reproducible inflammatory stimulus. Animals were assigned to treatment groups using a random number table, and the kinetic profiles of pro- and anti-inflammatory cytokines over a 72 h period are illustrated in Figure 10. Stigmasterol (**1**) treatment resulted in a significant modulation of early systemic mediators. Notably, the peak concentration of TNF-*α* at 2 h post-stimulation was significantly attenuated compared to the LPS-treated group (Figure 10A, * *p* < 0.05). Similarly, the levels of IL-1*β* exhibited a more rapid reduction over time, particularly during the 6 to 12 h interval (Figure 10B). Regarding IL-6, the stigmasterol-treated group showed an accelerated decline in cytokine concentrations, reaching baseline levels earlier than the untreated inflammatory control (Figure 10C). Furthermore, lower concentrations of IFN-*γ* were maintained throughout the initial 24 h observation period (Figure 10D). These results suggest that stigmasterol (**1**) exerts a suppressive effect on the magnitude of the systemic pro-inflammatory peak, validating its role as a potent immunomodulatory agent.

### 2.11. Cytokine Ratios and Immunomodulatory Balance

**Figure 11:**
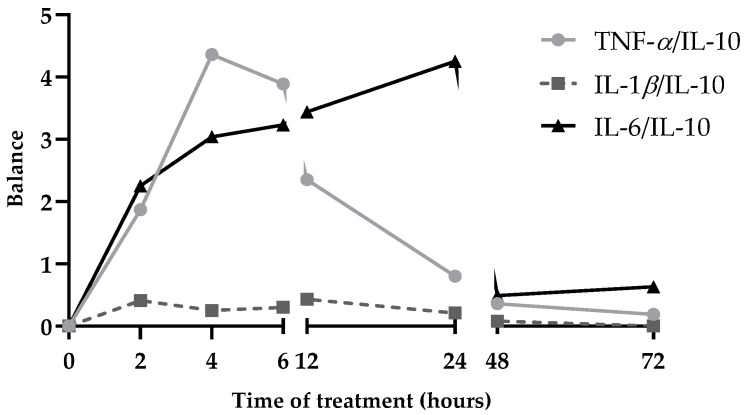
Kinetic analysis of the systemic immunomodulatory balance: ratios between pro-inflammatory mediators (TNF-*α*, IL-1*β*, IL-6) and the regulatory cytokine IL-10 following stigmasterol (**1**) treatment. Values below the threshold of 1.0 represent a shift toward the anti-inflammatory axis, indicating a predominant regulatory response that limits potential collateral tissue damage. Data are expressed as mean ± standard error of the mean (SEM) (*n* = 5). Statistical analysis was performed using two-way analysis of variance (ANOVA) to evaluate the effects of treatment and time, followed by Tukey’s post hoc test for multiple comparisons.

To evaluate the systemic balance between pro- and anti-inflammatory responses, the IL-6/IL-10, TNF-*α*/IL-10, and IL-1*β*/IL-10 ratios were calculated (Figure 11). Since IL-10 exerts significant regulatory functions that counteract the pro-inflammatory effects of IL-6 and TNF-*α*, a ratio value below 1.0 indicates a predominance of regulatory activity, which is generally associated with the protection of tissues from damage caused by persistent inflammation.

As illustrated in Figure 11, stigmasterol (**1**) treatment induced a temporal modulation of these mediators, favoring a transition toward a regulatory profile. While initial peaks were observed during the first 24 h reflecting the necessary activation of the acute inflammatory cascade, a marked mitigation of the pro-inflammatory dominance occurred thereafter. Notably, the TNF-*α*/IL-10 and IL-1*β*/IL-10 ratios remained consistently low or rapidly declined toward baseline, while the IL-6/IL-10 ratio exhibited a significant downward trend after 24 h, staying below the 1.0 threshold during the critical resolution phases (48–72 h).

This coordinated immunoregulatory effect appeared to limit excessive polarization and may restrict the over-recruitment of inflammatory cells, potentially ensuring that the cellular infiltrate remains sufficient for debris clearance without compromising the structural integrity of the epithelial margins [39,40]. In the context of tissue repair, the attenuation of early TNF-*α* signaling and the shortened persistence of IL-6 are associated with a reduced risk of collateral tissue damage, creating a favorable immunological shift that supports the early initiation of collagen synthesis within the wound bed.

Furthermore, the sustained elevation of IL-10 under stigmasterol (**1**) treatment highlights the activation of the resolution axis. While these findings strongly suggest an accelerated transition from the inflammatory to the proliferative phase, the specific signaling pathways governing the macrophage phenotypic switch in response to stigmasterol (**1**) remain a subject for future transcriptomic validation.

## 3. Discussion

### 3.1. Phytochemical Profile and Its Anti-Inflammatory and Antibacterial Dynamics

The selection of *B. bipinnata* for this study was based on its documented traditional medicinal use in Mexican ethnobotanical studies [5,20]. Consistent with these therapeutic applications, preliminary GC-MS analysis of the crude resin revealed a complex phytochemical profile dominated by terpenoids. This composition aligns with the chemical patterns reported for other species of the *Bursera* genus, which are typically characterized by high proportions of monoterpenes and triterpenes [35]. It is important to note, however, that while GC-MS is a robust screening tool, it does not provide unequivocal identification for all components; specifically, structural isomers such as the lupeol and 3-epilupeol pair exhibit nearly identical mass spectra and cannot be distinguished by GC-MS alone. Nevertheless, this class of secondary metabolites is extensively associated with anti-inflammatory, antimicrobial, and regenerative properties, suggesting that the resin’s ethnopharmacological efficacy stems from a multi-target synergy among its various components.

The anti-inflammatory activity observed in this study was assessed using the TPA-induced mouse ear edema model, an established approach for evaluating acute inflammation. TPA induces characteristic responses such as vasodilation and edema, primarily mediated by elevated intracellular cAMP levels and increased prostaglandin production [41]. The fact that *B. bipinnata* resin achieved an inhibition rate (49.08%) closely approaching that of indomethacin (55.76%) suggests a significant capacity to modulate these inflammatory pathways. This activity is hypothesized to involve the modulation of protein kinase C (PKC) or the inhibition of phospholipase A_2_ (PLA_2_), as the resin may influence the early expression of pro-inflammatory mediators. [42,43]. Although the anti-inflammatory potential of this species has been previously recognized, it is essential to consider that the presence and concentration of bioactive metabolites are subject to significant annual and seasonal fluctuations. Environmental factors and the specific timing of resin collection can substantially alter the chemical profile, potentially modifying the pharmacological potency of the material [43,44]. Therefore, analyzing biological activity each time these natural materials are collected is essential to accurately correlate phytochemical markers with the therapeutic effects observed in these complex matrices.

Regarding the antibacterial activity, the observed effects (MIC 125–250 µg/mL) are likely attributed to terpenoid components; however, this is considered secondary to the primary immunomodulatory effects. By potentially limiting the bacterial bioburden during the early inflammatory phase, the resin provides a protective environment that allows the potent regenerative effects of compounds like stigmasterol (**1**) to predominate without the interference of opportunistic infections [45,46]. These results indicate that, while not the primary driver of the healing process, the antibacterial properties may act as a complementary defensive barrier, potentially aiding in the prevention of opportunistic infections and thus supporting the overall regenerative environment promoted by the resin’s other bioactive constituents. Considering the established chemical profile and the observed moderate antibacterial activity, the focus of the investigation was directed toward identifying the specific constituents responsible for the wound-healing effects. This prioritization was further justified by the fact that many of the resin’s primary components have been previously evaluated for their anti-inflammatory potential, thereby emphasizing the importance of elucidating the direct role of these metabolites in tissue repair and regeneration.

### 3.2. Wound Healing Efficacy and Structure-Activity Relationship (SAR)

The therapeutic potential of *B. bipinnata* resin is particularly evident at higher concentrations (5.0 mg/mL), which appear to facilitate the transition from the inflammatory phase into the resolution stage of repair. This biological effect was primarily concentrated within the medium-polarity fraction (LR-F2), a crucial finding that served as the foundation for the bioactivity-guided isolation of the most potent constituents. While the reference drug, pirfenidone (PFD), is known to inhibit fibroblast proliferation during the early stages of healing [36,47], the metabolites concentrated in LR-F2 showed a more pronounced influence during the later proliferative and remodeling phases.

To determine the specific contribution of each isolate independent of their absolute mass, an equimolar concentration of 1 µM was selected for the second stage of the study. This approach is fundamental to evaluate molecular potency, as it ensures that the healing effects are compared based on a standardized molecular density per volume [47]. This distinction is revealing when compared to the initial evaluation of the crude resin and fractions, where PFD was applied at its therapeutic dose of 0.08 g/mL. The fact that the isolated terpenoids reached comparable healing rates while utilizing a significantly lower number of molecules provides definitive evidence of their high intrinsic activity.

The isolation of pure compounds from LR-F2 allowed for a specialized SAR analysis, particularly among the lupane-type triterpenoids (**2**, **3**, and **5**), which share a common pentacyclic skeleton. As summarized in Table 8, the functional group at the C-3 position is a critical determinant of biological potency.

A direct comparison between these molecules reveals that 3-epilupeol (**5**), which possesses a hydroxyl group in the *α*-configuration, exhibited superior efficacy compared to lupenone (**3**), where the hydroxyl is oxidized to a ketone [45,48,49]. This finding underscores the importance of a free, polar, and hydrogen bond-donating group at C-3 to trigger tissue repair pathways [48,49]. This is further evidenced by the reduction in activity observed when this position is esterified, as seen in lupeol acetate (**2**), which likely modulates lipophilicity but reduces specific molecular interactions.

It is important to emphasize that stigmasterol (**1**) was excluded from this structural SAR analysis. Despite showing the highest overall efficacy in the model, stigmasterol (**1**) possesses a stigmastane-type steroid skeleton. Since its core scaffold and biosynthetic origin are distinct from the pentacyclic lupane triterpenoids, its pharmacological activity is likely mediated through different molecular targets. The superior performance of stigmasterol (**1**) at 1 µM reinforces the conclusion that the isolation process successfully identified molecules with exceptional potency, capable of driving efficient tissue regeneration at low molecular concentrations.

### 3.3. Immunomodulatory Mechanism: Cytokine and Growth Factor Modulation

The histological progression observed aligns with the spatiotemporal modulation of cytokine profiles. The early attenuation of pro-inflammatory markers, coupled with a relative increase in IL-10, facilitated the accelerated tissue maturation identified in the histological sections. Within the signaling cascade, early TGF-*β*1 expression is known to promote macrophage recruitment and fibroblast activation [50,51]. A critical divergence emerged at 72 h: while stigmasterol (**1**) maintained a persistent elevation of TGF-*β*1, the resin-treated group exhibited a trend toward resolution. This suggests that the complex phytochemical matrix of the resin may provide a more regulated signaling environment, optimizing the transition to functional remodeling while potentially mitigating the risk of fibrosis [52,53].

The systemic immunomodulatory activity of stigmasterol (**1**), evaluated through the LPS-induced model, further supports its role in accelerated healing. The attenuation of the TNF-*α* peak prevents excessive extracellular matrix degradation, while the normalization of IL-6 limits collateral tissue damage [54]. These findings are consistent with previous reports identifying stigmasterol (**1**) as a potent inhibitor of the TLR4/NF-*κ*B signaling pathway. By preventing the translocation of NF-*κ*B to the nucleus, stigmasterol (**1**) effectively reduces the transcriptional activation of key pro-inflammatory genes, including TNF-*α* and IL-1*β* [55,56]. Furthermore, stigmasterol (**1**) has been characterized as a natural ligand for PPAR-*γ*, a nuclear receptor that exerts anti-inflammatory effects by antagonizing NF-*κ*B activity and promoting the polarization of M_2_ macrophages [57,58,59,60]. This molecular mechanism provides a robust explanation for the favorable cytokine ratios (IL-6/IL-10 and TNF-*α*/IL-10) and the selective preservation of IL-10 observed in our study.

Although systemic endotoxemia does not fully replicate local wound events, these data serve as supportive evidence of the compound’s intrinsic pharmacological potential to balance the immune response through specific molecular targets. Finally, this study represents a pioneering pharmacological evaluation of *B. bipinnata* resin using a murine excisional wound model. The selection of an acute wound model establishes a fundamental baseline of biological activity, providing robust proof-of-concept data that paves the way for future explorations in impaired-repair scenarios, such as chronic and diabetic wounds.

### 3.4. Limitations

The present study has several limitations. First, the experiments were performed in a model of acute (non-impaired) excisional wounds; therefore, the efficacy of stigmasterol (**1**) in chronic, diabetic, or infected wounds remains to be determined. Second, the sample size (*n* = 5 per group) is the minimum required for parametric statistics and should be confirmed in larger, independent cohorts. Third, the absence of in vitro validation, such as the use of primary cultures of keratinocytes or fibroblasts, limits the characterization of the direct cellular effects on proliferation and migration. Fourth, the lack of cutaneous pharmacokinetic data represents a significant gap, as the skin penetration and residence time of the isolated terpenoids after topical application have not yet been established. Fifth, the LPS-induced systemic inflammation model does not fully recapitulate the local cutaneous immune environment, and the systemic route used in that experiment differs from the topical route applied in the wound-healing model. Sixth, the purity of the isolated compounds, ranging from 92% to 97% by GC-MS area normalization, should be confirmed by orthogonal methods, such as HPLC-DAD or qNMR, before any pharmaceutical development. Eighth, the systemic immunomodulatory experiment employed oral administration of stigmasterol (**1**) rather than the topical route used in the excisional model. This methodological choice was dictated by the systemic nature of the LPS challenge, which precludes meaningful evaluation through cutaneous application. Consequently, the cytokine kinetics reported in Section 2.10 should be interpreted as supportive evidence of the intrinsic immunomodulatory capacity of stigmasterol (**1**) rather than as a direct mechanistic correlate of its topical wound-healing activity. A future study integrating microdialysis or matched topical/oral pharmacokinetic profiling will be required to formally bridge both compartments. Finally, the molecular mechanism by which stigmasterol (**1**) promotes wound healing was inferred from cytokine kinetics; direct demonstration of the proposed targets, including PPAR-*γ*, NF-*κ*B, and TLR4, will require dedicated mechanistic studies.

## 4. Materials and Methods

### 4.1. Resin Collection

The resin of *B. bipinnata* (DC.) Engl. was collected on 12 October 2022 from adult trees growing in Tepalcingo, located in the southern region of the “El Limón” Biological Station (18°31′52″ N; 98°56′15″ W), Morelos, Mexico, at an altitude of approximately 1300 m above sea level. The species was identified and authenticated by M.C. Gabriel Flores Franco (Center for Research in Biodiversity and Conservation, CIByC, Universidad Autónoma del Estado de Morelos, UAEM). The botanical name was verified using World Flora Online. A voucher specimen (No. 29019) was deposited in the HUMO Herbarium at CIByC, UAEM, Morelos, Mexico.

Fresh resin exudate was manually harvested from naturally occurring bark incisions of four individual adult trees. The collected samples were pooled into a single representative batch to minimize individual biological variability. This non-destructive collection method was performed as a single sampling event. The collection procedure complied with the Nagoya Protocol and the Mexican National Biodiversity Strategy. The resin was air-dried at room temperature under protected conditions for 72 h until reaching a constant weight, and it was subsequently stored in airtight containers, protected from light and humidity, until further processing.

### 4.2. Bioactivity-Guided Fractionation and Isolation of Bioactive Compounds

#### 4.2.1. Primary Fractionation

A bioactivity-guided fractionation strategy was employed to isolate the wound-healing constituents of *B. bipinnata* resin. Fifty grams (50.0 g) of crude resin were dissolved in dichloromethane (DCM) and adsorbed onto 800 g of silica gel (230–400 mesh), which was previously deactivated with 10% (*w*/*w*) distilled water. The deactivation procedure involved the gradual addition of water to the dry silica gel in a sealed glass container, followed by vigorous mechanical agitation until a homogeneous, free-flowing powder was obtained. The adsorbent was allowed to equilibrate for 24 h prior to column packing. This step was implemented to reduce the surface activity of the stationary phase, thereby preventing the excessive or irreversible retention of polar compounds and ensuring an efficient elution of the target constituents.

The resulting mixture was packed into a glass column and sequentially eluted with a gradient of solvents of increasing polarity: *n*-hexane (100%), *n*-hexane: DCM (1:1), DCM (100%), DCM: methanol (1:1), and methanol (100%). This stepwise polarity gradient allowed for the separation of constituents ranging from nonpolar to highly polar. Twenty-five fractions of 20 mL were collected for each solvent system. Their chemical profiles were monitored by thin-layer chromatography (TLC) using silica gel 60 *F*_254_ plates, visualized under UV light (254 and 365 nm) and derivatized with anisaldehyde, sulfuric acid reagent.

Fractions displaying similar TLC patterns were pooled to yield three primary groups: LR-F1 (26.45 g, 52.9% *w*/*w*) obtained with *n*-hexane, LR-F2 (18.12 g, 36.2% *w*/*w*) obtained with dichloromethane, and LR-F3 (4.23 g, 8.5% *w*/*w*) obtained with methanol. These fractions were evaluated in an in vivo wound-healing model, where LR-F2 exhibited the highest efficacy and was consequently prioritized for subsequent fractionation and isolation.

#### 4.2.2. Second Fractionation

To further resolve the bioactive constituents within LR-F2, a second open-column chromatography step was performed. An aliquot of 10.5 g was taken from the total LR-F2 fraction (18.12 g) and loaded onto 315 g of silica gel (230–400 mesh) deactivated with 10% distilled water (ratio 1:30 *w*/*w*). A gradient elution beginning with 100% *n*-hexane and progressively increasing the proportion of DCM (98:2, 95:5, 90:10, 80:20 *v*/*v*) was applied to increase the resolution of compounds with intermediate polarity. Eighteen fractions (50 mL each) were collected and monitored by TLC as previously described.

Based on their chromatographic similarity, these fractions were grouped into five subfractions with their respective yields relative to the initial crude resin: LR-F2-A (1.90 g, 6.55% *w*/*w*), LR-F2-B (1.17 g, 4.03% *w*/*w*), LR-F2-C (2.69 g, 9.11% *w*/*w*), LR-F2-D (1.79 g, 6.17% *w*/*w*), and LR-F2-E (2.49 g, 8.59% *w*/*w*). All subfractions were assessed in the wound-healing model; LR-F2-A was prioritized for final compound isolation due to its chemical consistency and biological potency.

### 4.3. Structural Identification of Compounds

#### 4.3.1. Compound Isolation and Final Purification

The isolation of bioactive constituents from the prioritized subfraction was performed using a bioactivity-guided fractionation approach. An aliquot of 0.3053 g of the LR-F2-A subfraction was subjected to isocratic Flash Column Chromatography (FCC) on a silica gel 60 stationary phase (70–230 mesh), which had been previously deactivated with 10% (*w*/*w*) distilled water to optimize the resolution of structural isomers. Elution was carried out using a low-polarity isocratic system of *n*-hexane: ethyl acetate (98:2, *v*/*v*), resulting in the successful isolation of five main terpenoid constituents.

The yields and relative abundances (*w*/*w*) based on the initial crude resin (50.0 g) were: stigmasterol (**1**, 0.0221 g, 0.47% *w*/*w*), obtained as white needle-like crystals; lupeol acetate (**2**, 0.0101 g, 0.21% *w*/*w*), as a white crystalline solid; lupenone (**3**, 0.0194 g, 0.40% *w*/*w*), as a white amorphous powder; caryophyllene oxide (**4**, 0.0138 g, 0.27% *w*/*w*), as a colorless crystalline solid; and 3-epilupeol (**5**, 0.0352 g, 0.75% *w*/*w*), as a white crystalline solid.

Due to limited mass recovery, compounds **2** and **4** were utilized exclusively for structural elucidation and purity verification. Conversely, compounds **1**, **3**, and **5** were obtained in sufficient yields to conduct the in vivo pharmacological evaluation. These compounds were incorporated into a topical treatment protocol, administered daily for the first three days post-wounding (*n* = 5), followed by a longitudinal monitoring period until day 14 to assess their respective healing potencies.

#### 4.3.2. Structural Identification and Purity Determination

The chemical identity of the isolated compounds was confirmed through spectroscopic and spectrometric analysis. ^1^H and ^13^C NMR spectra were recorded on a Bruker Avance III 400 MHz spectrometer (and a 300 MHz system for compounds **4** and **5**) using CDCl_3_ as the solvent and TMS as an internal standard. Characterization was supported by the following diagnostic signals: stigmasterol (**1**) showed signals at *δ*_H_ 3.52 (m, H-3), 5.35 (br d, *J* = 5.2 Hz, H-6), 5.01 (dd, *J* = 15.2, 8.7 Hz, H-22), and 5.15 (dd, *J* = 15.2, 8.6 Hz, H-23); lupeol acetate (**2**) exhibited signals at *δ*_H_ 4.47 (dd, *J* = 10.8, 5.4 Hz, H-3), 4.56 (m, H-29a), 4.68 (m, H-29b), and 2.04 (s, CH_3_CO); lupenone (**3**) displayed signals at *δ*_H_ 2.41–2.49 (m, H-2), 4.57 (m, H-29a), and 4.69 (m, H-29b), with a characteristic carbonyl signal at *δ*_C_ 218.2 (C-3); caryophyllene oxide (**4**) was identified by signals at *δ*_H_ 1.20 (s, H-12), 2.88 (dd, *J* = 10.7, 4.3 Hz, H-5), and 4.86–4.98 (m, H-15); and 3-epilupeol (**5**) presented diagnostic signals at *δ*_H_ 3.39 (br s, H-3*α*), 2.38 (td, *J* = 11.0, 5.8 Hz, H-19), 4.56 (d, *J* = 2.3 Hz, H-29a), and 4.68 (d, *J* = 2.3 Hz, H-29b).

Mass spectrometry data were acquired using an Agilent 5975C GC/MSD system. Purity was determined by GC-MS area normalization and corroborated by analyzing the signal-to-noise ratio in the NMR spectra. The estimated purity for all isolates ranged from 92% to 97%, representing the technical limit for the separation of structural isomers in *Bursera* resins. Characterization data and complete spectra are provided in the Supplementary Materials (Tables S6–S10 and Figures S2–S21).

#### 4.3.3. Gas Chromatography Mass Spectrometry (GC-MS) Analysis of *Bursera bipinnata* Resin

Gas chromatography mass spectrometry (GC-MS) analysis of *B. bipinnata* resin was performed using a 7890B gas chromatograph coupled with a 5977A mass selective detector (Agilent Technologies, Santa Clara, CA, USA) via Agilent MassHunter GC-MS acquisition software (version B.07.02.1938). The analysis was conducted at the Institute of Chemistry, National Autonomous University of Mexico (UNAM). The separation of the compounds was achieved using an HP-5MS capillary column (30 m × 0.25 mm × 0.25 µm) with ultrapure helium as the carrier gas at a constant flow rate of 60 mL h^−1^. A 1 µL sample was injected in splitless mode. The oven temperature was initially set at 60 °C and maintained for 2 min, followed by a temperature increase to 285 °C at a rate of 5 °C min^−1^, with a final hold at 285 °C for 3 min. The mass spectrometer was operated in electron ionization (EI) mode with an ion source temperature of 230 °C, a quadrupole temperature of 150 °C, and an electron energy of 70 eV. The electron multiplier voltage was set to 1859 V, and data acquisition was performed in full-scan mode using the SCAN GENERAL MLS.M method. The resulting mass spectra were processed using the Mayra L.S. method and compared against the National Institute of Standards and Technology (NIST) mass spectral library for compound identification. The data file was saved as LM*Bb* 1.D, and the analysis was completed on August 18, 2023, at 9:15 p.m., by M. en C. Mayra Leon Santiago.

### 4.4. TPA-Induced Ear Edema (Anti-Inflammatory Activity)

#### 4.4.1. Rationale of the Model

The TPA-induced ear edema model is a robust and widely used assay for evaluating the acute anti-inflammatory potential of natural products. Topical application of 12-O-tetradecanoylphorbol-13-acetate (TPA) triggers a rapid inflammatory response primarily mediated by the activation of Protein Kinase C (PKC). This activation leads to the release of pro-inflammatory mediators, such as prostaglandins and leukotrienes, resulting in increased vascular permeability, vasodilation, and neutrophil recruitment. By measuring the reduction in edema (weight and thickness) in treated versus untreated ears, the intrinsic anti-inflammatory efficacy of the *B. bipinnata* resin can be quantified [61,62].

#### 4.4.2. Experimental Animals–TPA Induced Ear Edema Model

Male BALB/c mice (25–30 g) were obtained from the animal facility of the Facultad de Medicina, Universidad Autónoma del Estado de Morelos (UAEM). The animals were housed under controlled environmental conditions (22 ± 2 °C; 12 h light/dark cycle), with free access to standard chow and water. All the experimental protocols adhered to the Official Mexican Standard NOM-062-ZOO-1999 [63], which establishes technical specifications for the care and use of laboratory animals and is in accordance with the Guide for the Care and Use of Laboratory Animals (8th edition, National Research Council, USA). Ethical principles, including the use of the minimum number of animals required to obtain statistically valid results, were followed to ensure animal welfare. The experimental procedures conducted in this study were reviewed and approved by the Institutional Committee for the Care and Use of Laboratory Animals (CICUAL) of the university under protocol number PRO-CICUAL-006/2025.

#### 4.4.3. Edema Induction and Treatment

Acute inflammation was induced by the topical application of 12-O-tetradecanoylphorbol-13-acetate (TPA, 2.5 μg/ear) dissolved in acetone (0.125 mg/mL). A total volume of 20 μL of the TPA solution was applied to the right ear of each mouse, distributed as 10 μL on the inner surface and 10 μL on the outer surface to ensure full tissue exposure. Ten minutes after induction, 10 μL of each test sample was applied topically. Experimental treatments included three doses of *B. bipinnata* resin (0.03, 0.10, and 0.30 mg/ear), whereas the vehicle group received acetone and the positive control group received indomethacin (0.10 mg/ear). All substances were dissolved in acetone to ensure uniform absorption and standardized experimental conditions across all groups.

#### 4.4.4. Experimental Groups

The animals were randomly distributed into five experimental groups (*n* = *5* per group). The sample size was determined as the minimum number of individuals required to achieve sufficient statistical power for parametric analysis while strictly adhering to the 3Rs principle (Replacement, Reduction, and Refinement) for the ethical use of animals in research. This reduction strategy aims to minimize the total number of experimental units without compromising the reproducibility or the scientific validity of the inflammatory assessment. The groups were assigned as follows:

Group 1: VC—Vehicle Control (acetone + TPA)

Group 2: Indomethacin-Positive Control (indomethacin 0.10 mg/ear + TPA)

Group 3: *B. bipinnata* resin at 0.03 mg/ear + TPA

Group 4: *B. bipinnata* resin at 0.10 mg/ear + TPA

Group 5: *B. bipinnata* resin at 0.30 mg/ear + TPA

#### 4.4.5. Measurement of Inflammation and Statistical Analysis

Four hours after TPA application, mice were euthanized and ear edema was quantified. Thickness was measured using a digital micrometer (Mitutoyo^®^ Series 293, Kawasaki, Japan). Additionally, circular biopsies (7 mm) were weighed. The percentage of anti-inflammatory inhibition was calculated using the following formula:

(1) $$\%\;\mathrm{of}\;\mathrm{inhbition}\;=\frac{w_{\mathrm{control}\;}-\;w_{\mathrm{Treatment}\;}}{w_{\mathrm{control}}}\;\times \;100$$

where $w_{\mathrm{control}}$ represents the difference in weight between the left ear treated with the vehicle and the right ear (control), and $w_{\mathrm{Treatment}}$ represents the difference in weight between the left ear of each group treated with the different treatments and the right ear (control). Data were analyzed using one-way ANOVA followed by Dunnett’s post hoc test to compare all experimental groups against both the negative control (for efficacy) and the positive control (indomethacin, for relative potency).

### 4.5. Wound-Healing Excision Model

#### 4.5.1. Experimental Animals–Wound Healing Model

Male BALB/c mice (20–25 g) were obtained from the animal facility of the Facultad de Medicina, Universidad Autónoma del Estado de Morelos (UAEM). The animals were maintained under standard laboratory conditions (22 ± 2 °C; 12 h light/dark cycle), with food and water provided ad libitum. All procedures were conducted in strict compliance with the Official Mexican Standard NOM-062-ZOO-1999 [63], which outlines the technical specifications for the care and use of laboratory animals. Additionally, the experiments conformed to the ethical guidelines described in the Guide for the Care and Use of Laboratory Animals (8th edition, National Research Council, USA). The number of animals used was minimized and justified statistically to reduce suffering. The experimental procedures conducted in this study were reviewed and approved by the Institutional Committee for the Care and Use of Laboratory Animals (CICUAL) of the university under protocol number PRO-CICUAL-006/2025.

#### 4.5.2. Surgical Procedure for Skin Incisions

A dermal wound model was established following a standardized protocol to ensure consistency and minimize animal suffering [64,65]. The dorsal region of each mouse was shaved with an electric clipper and disinfected with 70% ethanol to remove hair and reduce microbial contamination in the wound area. Animals were then anesthetized via inhalation of isoflurane, with induction performed at 3.0% *v*/*v* in 100% *O*_2_ and maintenance at 1.5–2% *v*/*v*, until the complete loss of the pedal reflex and stable spontaneous breathing were achieved.

A sterile 5.0 mm biopsy punch [66] was used to create a full-thickness skin incision, which was carefully performed using sterile forceps and scissors. The wounds were left open to simulate physiological conditions, and daily macroscopic examinations were conducted to monitor for clinical signs of secondary infection, such as erythema, exudate, and edema.

#### 4.5.3. Experimental Groups and Treatments

The mice were randomly assigned to six groups (*n* = 5 per group):

Group 1: NC—negative control (untreated)

Group 2: VC—vehicle control (Tween 80)

Group 3: PC—positive control (Pirfenidone 0.08 g/mL, Kitoscell^®^)

Group 4: *B. bipinnata* resin at 1.2 mg/mL

Group 5: *B. bipinnata* resin at 2.5 mg/mL

Group 6: *B. bipinnata* resin at 5.0 mg/mL

Topical treatments (20 μL per wound) were applied once daily for the first three days. All the animals were monitored for general health and wound conditions throughout the 12-day experimental period.

#### 4.5.4. Wound Contraction Assessment

Wounds were photographed on days 0, 2, 5, 7, 9, and 12 using a Canon T6 camera with an 18–55 mm lens (Canon Inc., Tokyo, Japan) under standardized lighting conditions and a fixed distance. [27,67]. Wound areas were quantified using ImageJ software (version win64) to ensure accuracy and reproducibility. As an alternative validation technique, the wound area was also assessed using a transparent grid composed of 1 mm^2^ squares superimposed over the wound to manually count the affected area [68]. The percentage of wound contraction was calculated with the following formula:

(2) $$Wound\;contraction\;percentage\;=\left[\frac{\left(A_{0}-A_{F}\right)}{A_{0}}\right]\times 100$$

where $A_{0}$ is the initial area, corresponding to the day of the surgical procedure, and where $A_{F}$ is the final area on the specified days [69].

#### 4.5.5. Mean Wound Healing Time

A separate follow-up was conducted to determine the mean wound healing time (MWHT) for each treatment group [8,27]. The animals were monitored daily until complete macroscopic wound closure was observed, which was defined as full re-epithelialization without the presence of a scab or exudate. The number of days required for complete healing was recorded for each mouse, and the MWHT was calculated as the average number of days for each treatment group. The formula used to calculate the MWHT was as follows:

(3) $$MWHT=\frac{\sum \left(Days\;to\;complete\;healing\;for\;each\;mouse\;in\;the\;group\right)}{Number\;of\;mice\;in\;the\;group}$$

#### 4.5.6. Macroscopic Evaluation of Wounds

Wounds were examined visually daily for any signs of infection, including erythema, exudate, and edema, as well as hemorrhage, scab formation, and the presence of granulation tissue [70]. The inspections were conducted under standardized lighting conditions to minimize potential visual bias. A magnifying lens was used to enhance the visibility of subtle changes in the wound, ensuring thorough and accurate assessments. Photographs were taken at consistent angles and fixed distances to maintain uniformity and allow for accurate comparisons of wound progression over time. Additionally, qualitative parameters, such as color, texture, and the presence of necrotic tissue in the wound bed, were systematically documented. These observations complemented the quantitative measurements of wound contraction, providing a comprehensive evaluation of the healing process.

### 4.6. Histological Analysis of Cutaneous Wound Healing

Skin samples were collected from the wound area at predetermined time points (days 2, 5, 7, 9, and 12 post-wounding) [51]. Animals were euthanized according to institutional ethical guidelines, and full-thickness skin specimens encompassing the wound site and surrounding healthy tissue were carefully excised. Tissue samples were immediately fixed in 10% neutral-buffered formalin for 24–48 h at room temperature. Following fixation, specimens were dehydrated through a graded ethanol series, cleared in xylene, and embedded in paraffin. Paraffin-embedded tissues were sectioned at 5 μm using a rotary microtome, mounted on glass slides, and stained with hematoxylin and eosin (H&E) following routine protocols [71].

Histological sections were examined using a light microscope (Olympus BX53, Olympus Corporation, Tokyo, Japan) at 10×, 20×, and 40× magnifications. Representative micrographs were captured using a digital imaging system. The analysis focused on key morphological parameters, including epidermal continuity, inflammatory cell infiltration, fibroblastic proliferation, and angiogenesis [72]. To ensure the objectivity of the assessment, histological scoring was performed by two independent, blinded observers. The semi-quantitative evaluation was based on a four-point scale: (-) Absence; (+) Mild/Scant (scattered cells or minimal features, <25% of the field); (++) Moderate (consistent presence, 25–50% of the field); and (+++) Marked/Complete (extensive presence or dense features, >50% of the field). Discrepancies between observers were resolved by consensus. Histological features were interpreted in accordance with the classical phases of cutaneous wound healing [51].

### 4.7. Quantification of TGF-β1, IL-6, and IL-10 in Skin Wound Tissue

To further investigate the molecular mechanisms underlying the wound-healing activity observed for the resin of *B. bipinnata* and the isolated compound stigmasterol (**1**), the levels of transforming growth factor beta-1 (TGF-*β1*), interleukin-6 (IL-6), and interleukin-10 (IL-10) were determined directly in wounded skin tissue. These cytokines play central roles in regulating inflammatory resolution, fibroblast activation, and the transition to the proliferative phase. Male BALB/c mice (20–25 g) were maintained under controlled environmental conditions (22 ± 2 °C, 12 h light/dark cycle) with free access to standard rodent chow and water. All experimental procedures were conducted in accordance with the Mexican Official Standard NOM-062-ZOO-1999 [63] and international guidelines for laboratory animal welfare.

Excisional wounds were generated following the surgical procedure previously described. Briefly, the dorsal region of each mouse was shaved with an electric clipper and disinfected with 70% ethanol. Animals were anesthetized via inhalation of isoflurane (induction at 3.0% *v*/*v* in 100% *O_2_*, maintenance at 1.5–2% *v*/*v*) until complete loss of the pedal reflex and stable spontaneous breathing were achieved. A full-thickness excisional wound was then created using a sterile 5.0 mm biopsy punch. Topical treatments were applied once daily for three consecutive days: *B. bipinnata* resin (5.0 mg/mL) and stigmasterol (**1**) (1 μM) in a volume of 20 μL per lesion. Skin samples were collected at 24 h and 72 h after the first treatment application. For each time point, four animals per treatment group were randomly selected for cytokine determination. Skin biopsies were obtained from the wound site, rapidly frozen in liquid nitrogen, and stored at −80 °C. Frozen tissue samples were homogenized in cytoplasmic lysis buffer supplemented with protease inhibitors (Roche Diagnostics, Mannheim, Germany) using a FastPrep system (Q-Biogene, Solon, OH, USA), followed by centrifugation at 5000× *g* for 10 min. The resulting supernatants were used for cytokine determination. In addition to TGF-*β*1 (Cat. No. 88-8350-22), the local concentrations of IL-6 (Cat. No. 88-7064-22) and IL-10 (Cat. No. 88-7105-22) were quantified using mouse-specific sandwich ELISA kits (Invitrogen, Thermo Fisher Scientific, Waltham, MA, USA) according to the manufacturer’s instructions. All assays were performed in duplicate. Total protein concentration in each tissue homogenate was determined using the Pierce™ BCA Protein Assay Kit (Cat. No. 23225). Cytokine levels were normalized to total protein and expressed as picograms of cytokine per milligram of total protein (pg/mg protein). Statistical differences were evaluated using one-way analysis of variance (ANOVA) followed by Tukey’s multiple comparison test (* *p* < 0.05).

### 4.8. LPS-Induced Systemic Inflammation Model

To further evaluate the systemic immunomodulatory potential of the isolated compounds and their influence on the inflammatory response, an LPS-induced systemic model was employed. While wound healing is a localized process, it is modulated by systemic immune signaling. The LPS-induced systemic inflammation model was therefore utilized as supportive evidence to dissect the cytokine-level patterns associated with the immunomodulatory effects observed in the wound-healing assay. Male BALB/c mice (8–10 weeks old, 25–30 g) were obtained from the institutional animal facility and randomly assigned to the experimental groups before being acclimated for at least one week prior to experimentation. Animals were housed under controlled environmental conditions (22 ± 2 °C, 50–60% humidity, 12 h light/dark cycle) with free access to standard chow and water. All experimental procedures were conducted in accordance with the ARRIVE guidelines and were approved by the Institutional Animal Care and Use Committee. To induce systemic inflammation, mice received a single intravenous injection of lipopolysaccharide (LPS) from *Escherichia coli* 0111:B4 at a dose of 2.0 mg/kg body weight, dissolved in sterile physiological saline and administered via the tail vein. This dose and route were selected based on their ability to elicit a robust and reproducible systemic inflammatory response characterized by rapid induction of pro- and anti-inflammatory cytokines without causing excessive mortality [73]. Stigmasterol (**1**), identified as a major bioactive constituent of *B. bipinnata* resin, was administered orally at a dose of 20 mg/kg. The compound was suspended in an appropriate vehicle and administered once daily for three consecutive days prior to LPS challenge to ensure adequate systemic exposure at the time of inflammation induction. Control groups received the corresponding vehicle alone. Blood samples were collected at predefined time points (0, 2, 6, 12 and 24 h) following LPS administration to evaluate the kinetics of cytokine production [74]. At each time point, animals were anesthetized with isoflurane and blood was obtained by cardiac puncture. Samples were allowed to clot at room temperature and subsequently centrifuged at 3000× *g* for 10 min at 4 °C to obtain serum, which was stored at −80 °C until analysis. Serum concentrations of TNF-*α*, IL-1*β*, IL-6, IFN-*γ* and IL-10 were quantified using commercially available mouse-specific sandwich enzyme-linked immunosorbent assay (ELISA) kits, following the manufacturer’s instructions. All samples and standards were analyzed in duplicate. Absorbance was measured at 450 nm using a microplate reader, and cytokine concentrations were calculated from standard curves generated for each analyte [56]. The ratios between IL-6 and IL-10, and TNF-*α* and IL-10 represent a key mechanism in the regulation of the immune and inflammatory response. The IL-6/IL-10 and TNF-*α*/IL-10 balances were used as indicators of inflammatory balance. Values > 1 indicate a predominance of pro-inflammatory activity, whereas values < 1 suggest a predominance of anti-inflammatory activity (higher IL-10 relative to IL-6 or TNF-*α*). A value close to 1 reflects a more balanced cytokine profile. Data are expressed as mean ± standard error of the mean (SEM). Statistical analysis was performed using two-way analysis of variance (ANOVA) to evaluate the effects of treatment and time, followed by Tukey’s post hoc test for multiple comparisons. Differences were considered statistically significant at * *p* < 0.05.

The shift from the topical route used in the excisional wound model to oral administration in the LPS-induced systemic inflammation model was deliberate and methodologically justified. The excisional model is designed to assess local tissue repair, where topical application maximizes the concentration of the active compound at the site of injury and reproduces the traditional ethnomedicinal use of *B. bipinnata* resin as a poultice. In contrast, the LPS challenge induces a generalized endotoxaemic response in which the target organs (liver, spleen, vascular endothelium and circulating leukocytes) are anatomically distant from the skin; topical delivery would therefore be unable to ensure reproducible systemic exposure of stigmasterol (**1**) at the relevant compartments. Oral administration, by contrast, has been extensively validated for stigmasterol and other phytosterols, which exhibit measurable intestinal absorption (~5–10%) and a plasma half-life sufficient to achieve steady-state systemic concentrations after repeated dosing [75,76,77,78,79]. The 20 mg/kg/day regimen administered for three consecutive days before the LPS challenge was selected to ensure adequate systemic exposure at the time of immune activation, in line with previous pharmacological studies of stigmasterol in murine models of inflammation [76,77]. Accordingly, the two routes serve complementary, not interchangeable, pharmacological purposes: the topical route addresses the local cutaneous efficacy claimed by traditional use, whereas the oral route is required to demonstrate the intrinsic systemic immunomodulatory capacity of the compound, independently of the mechanical and barrier-related variables of the wound model. This complementary design is consistent with current recommendations for the pharmacological characterization of natural products with dual local and systemic relevance.

### 4.9. Antibacterial Activity (MTT Assay)

#### 4.9.1. Strains and Inoculum Preparation

The antibacterial activity of *B. bipinnata* resin was evaluated against six bacterial strains: *Escherichia coli* (ATCC 8739), *Streptococcus pyogenes* (ATCC 19615), *Pseudomonas aeruginosa* (clinical isolate), *Staphylococcus aureus* (ATCC 6538), *Salmonella typhimurium* (ATCC 14028), and methicillin-resistant *Staphylococcus aureus* (MRSA, ATCC 43300). The bacterial strains were grown under aerobic conditions at 37 °C. Inocula were prepared by suspending 3–5 colonies of similar morphology in 4–5 mL of sterile 0.85% saline. The suspensions were adjusted to a turbidity equivalent to a 0.5 McFarland standard (approximately 1–2 × 10^8^ CFU/mL) and further diluted to a final concentration of 5 × 10^5^ CFU/mL for use in microdilution assays [78].

#### 4.9.2. Resin Formulation and Control Preparation

The resin of *B. bipinnata* was dissolved under sterile conditions in a mixture consisting of 500 µL of Tween 80, 100 µL of DMSO, and 400 µL of sterile 0.85% saline. No filtration was required because of its complete solubility. This formulation was serially diluted in Mueller-Hinton broth (MHB) to prepare final test concentrations ranging from 500 to 3.9 µg/mL. The negative controls included vehicle controls prepared as follows: 200 µL of Tween 80 + 800 µL of saline and 100 µL of DMSO + 900 µL of saline. These factors were confirmed not to interfere with bacterial growth. Gentamicin (Sigma-Aldrich) was used as a positive control and was tested at concentrations ranging from 0.08 to 10 µg/mL. A sterility control (MHB only) was included in each assay.

#### 4.9.3. Broth Microdilution Assay

The antibacterial activity was first assessed using the broth microdilution method following the guidelines of the Clinical and Laboratory Standards Institute [79,80]. Serial dilutions of the resin (500–3.9 µg/mL) were prepared in Mueller-Hinton broth (MHB) and added to 96-well microplates (final well volume: 200 µL). Then, 100 µL of bacterial inoculum (5 × 10^5^ CFU/mL) was added. The plates were incubated statically at 37 °C for 24 h. The minimum inhibitory concentration (MIC) was defined as the lowest concentration at which visible bacterial growth was completely inhibited.

#### 4.9.4. MTT Bacterial Viability Assay

To complement and confirm the microdilution results, bacterial viability was assessed using a modified 3-(4,5-dimethylthiazol-2-yl)-2,5-diphenyltetrazolium bromide (MTT) colorimetric assay, which detects metabolically active bacteria [79,80]. After 24 h of incubation, 10 µL of MTT solution (0.4 mg/mL) was added to each well, followed by incubation for an additional 4 h at 37 °C. Viable bacteria reduce yellow tetrazolium salt to purple formazan, which is quantified by measuring the absorbance at 570 nm (with a reference wavelength of 630 nm) using a GloMax Multidetector System (Promega, Madison, WI, USA). This assay was used in parallel with the broth microdilution method to provide a quantitative measure of bacterial inhibition based on metabolic activity, enhancing the sensitivity and interpretability of the results. The percentage of inhibition was calculated using the following formula:

(4) $$Inhibition\;\left(\%\right)=1-\left(\frac{Abs\;treated}{Abs\;control}\right)\;\times \;100$$

where *Abs treated* is the absorbance of the treated wells and *Abs control* is the absorbance of the negative control wells.

A decrease in absorbance indicated the inhibition of bacterial growth. This colorimetric method is based on the reduction in yellow tetrazolium salt (MTT) to purple formazan by metabolically active cells, providing an indirect but reliable measurement of bacterial viability [81,82]. All the results are expressed as the means ± standard deviations. The inclusion of technical and biological replicates ensured statistical robustness and reproducibility.

### 4.10. Dermal Toxicity

To evaluate the irritation potential of the *B. bipinnata* resin-based treatment, an acute dermal irritation test was performed according to the OECD Guideline 404 [83]. For this assay, three adult BALB/c mice (*n* = 5) were utilized. The dorsal skin was shaved 24 h prior to the experiment to ensure the absence of pre-existing lesions. A volume of 0.5 mg/mL of the resin solution (standardized at 0.50 mg/mL in acetone) was applied to a designated 6 cm^2^ area of the intact skin and covered with a semi-occlusive patch for a 4 h exposure period. After patch removal, the site was gently cleaned with distilled water.

Dermal reactions, specifically erythema and edema, were scored at 1, 24, 48, and 72 h after the removal of the patch, according to the Draize scale (0–4 for erythema and 0–4 for edema). The Primary Irritation Index (PII) was calculated based on the sum of the scores divided by the number of observation points. A PII value of 0.00 was obtained for all animals, as no signs of erythema or edema were observed at any time point during the observation period. These findings categorize the resin as non-irritant under the tested conditions, providing supportive evidence for its safety in topical applications and aligning with its traditional medicinal use in Mexico, where the resin is applied directly to skin lesions without reported adverse effects.

### 4.11. Statistical Analysis

Statistical analysis was performed using GraphPad Prism software version 8.0.1 (GraphPad Software, San Diego, CA, USA) for Windows 10. All experiments were conducted in quintuplicate, and data are presented as the mean ± standard error of the mean (SEM). Data normality was verified using the Shapiro–Wilk test prior to performing one-way analysis of variance (ANOVA). When the normality assumption was met, Dunnett’s post hoc test was applied for pairwise comparisons between the experimental groups and the control. In cases where data transformation was necessary to satisfy the assumptions of parametric statistics, the appropriate mathematical transformation was applied. A *p*-value of less than 0.05 (* *p* < 0.05) was considered statistically significant. This analytical approach ensures a robust framework for the precise interpretation of the experimental data [84,85].

## 5. Conclusions

This study provides robust pharmacological evidence validating the traditional use of *B. bipinnata* resin in wound management. The results demonstrate that the crude resin possesses a significant dual action, acting as a potent anti-inflammatory and wound-healing agent, complemented by supportive antibacterial activity. The wound-healing efficacy was successfully traced back to its isolated constituents, specifically stigmasterol (**1**), 3-epilupeol (**5**), and lupenone (**3**), which significantly accelerated wound closure and promoted advanced tissue regeneration characterized by enhanced re-epithelialization and organized collagen deposition. A central finding of this investigation is the exceptional intrinsic potency of the isolated compounds. Mechanistically, these results are associated with a coordinated immunomodulation where stigmasterol (**1**) functions as a key regulator of the inflammatory-to-proliferative transition. This effect is likely mediated through the modulation of the TLR4/NF-κB and PPAR-*γ* signaling pathways, resulting in the systemic and local attenuation of pro-inflammatory cytokines (TNF-*α*, IL-1*β*, and IL-6) and the preservation of IL-10-mediated anti-inflammatory signaling. Furthermore, the persistent local expression of TGF-*β*1 within the wound microenvironment confirms the role of these metabolites in fibroblast activation and extracellular matrix synthesis. The results obtained in the LPS-induced systemic inflammation model further validate the intrinsic immunomodulatory capacity of stigmasterol (**1**), demonstrating its potential to balance the cytokine cascade beyond the localized injury site. To the best of our knowledge, this investigation provides the first in vivo pharmacological evidence in a murine excisional model regarding the specific and active role of stigmasterol (**1**) in the complex process of cutaneous tissue repair. Based on these findings, a local concentration of 1 μM for isolated markers or 5.0 mg/mL for standardized resin extracts is suggested as a fundamental baseline for the development of future topical pharmaceutical formulations. Collectively, these results reinforce the ethnopharmacological relevance of *B. bipinnata* resin as a promising source of bioactive markers for the development of standardized wound-healing treatments.

## Figures and Tables

**Table 1:** Anti-inflammatory activity of *B. bipinnata* resin in the TPA-induced mouse ear edema model.

Treatment	mg/Ear	Inhibition of Inflammation (%) ± SEM
NC	-	-
Vehicle	-	0.00
Indomethacin	0.10	55.76 ± 2.40 *
*Bb* resin	0.03	12.67 ± 1.65 *
	0.10	46.66 ± 3.06 *
	0.30	49.08 ± 1.03 *

Results are expressed as the mean ± SEM (*n* = 5). The Vehicle group represents the application of acetone exclusively, serving as a non-irritation control. The NC (Negative Control) group corresponds to the application of TPA dissolved in acetone and was used as the reference for maximum inflammation (0% inhibition). * *p* < 0.05 indicates statistically significant differences compared with the NC (Negative Control) group. Statistical significance was determined by one-way ANOVA followed by Dunnett’s post hoc test.

**Table 2:** Minimum Inhibitory Concentration (MIC) values of *B. bipinnata* resin against clinically relevant bacterial strains.

Treatments	MIC (µg/mL)					
	*E*.*c*	*S*.*p*	*P*.*a*	*S*.*a*	*S*.*t*	*S*.*a* MRSA
*B. bipinnata* resin	NE	125	NE	NE	250	NE
Gentamicin	0.32	≤0.32	0.62	0.16	0.32	10.0

*E*.*c*: *Escherichia coli*; *S*.*p*: *Streptococcus pyogenes*; *P*.*a*: *Pseudomonas aeruginosa*; *S*.*a*: *Staphylococcus aureus*; *S*.*t*: *Salmonella typhimurium*; *S*.*a* MRSA: methicillin-resistant Staphylococcus aureus. S/E: No effect; indicates that no antibacterial activity was detected up to the maximum evaluated concentration of 500 µg/mL.

**Table 3:** Percentage of *B. bipinnata* resin-induced wound contraction at different doses in the skin wound model.

Days	NC	PFD (0.08 g/mL)	Tween 80	*Bb* Resin (1.2 mg/mL)	*Bb* Resin (2.5 mg/mL)	*Bb* Resin (5.0 mg/mL)
	(%)					
0	00.00 ± 0.00	00.00 ± 0.00	00.00 ± 0.00	00.00 ± 0.00	00.00 ± 0.00	00.00 ± 0.00
2	03.59 ± 1.04	59.68 ± 3.59 *	11.63 ± 3.78 *	14.76 ± 2.72 *	20.38 ± 2.29 *	36.55 ± 2.17 *
5	11.14 ± 2.64	72.67 ± 6.80 *	25.14 ± 1.23 *	38.76 ± 2.44 *	59.53 ± 2.13 *	77.24 ± 2.40 *
7	23.43 ± 2.64	82.23 ± 6.70 *	43.79 ± 3.40 *	61.04 ± 2.86 *	92.63 ± 0.56 *	87.16 ± 2.20 *
9	57.85 ± 2.59	94.29 ± 3.37 *	61.65 ± 2.48	76.70 ± 0.86 *	97.19 ± 0.75 *	97.48 ± 0.40 *
12	78.37 ± 2.36	99.83 ± 2.62 *	84.80 ± 2.49 *	90.07 ± 0.44 *	99.08 ± 0.26 *	99.63 ± 0.10 *

Data are expressed as the mean ± SEM (*n* = 5). * *p* < 0.05 indicates a significant difference compared to the negative control (NC) group, as determined by one-way ANOVA followed by Dunnett’s post hoc test. Animals were assigned to experimental groups through a randomization process using a random number table, and treatments were topically administered daily only during the first three days post-wounding.

**Table 4:** Percentage of wound contraction caused by *B. bipinnata* resin fractions in the skin wound model.

Days	NC	PFD (0.08 g/mL)	Tween 80	LR-F1 (5.0 mg/mL)	LR-F2 (5.0 mg/mL)	LR-F3 (5.0 mg/mL)
	(%)					
0	00.00 ± 0.00	00.00 ± 0.00	00.00 ± 0.00	00.00 ± 0.00	00.00 ± 0.00	00.00 ± 0.00
2	03.59 ± 1.04	59.68 ± 3.59 *	11.63 ± 3.78 *	11.56 ± 2.22 *	30.05 ± 1.72 *	14.04 ± 1.34 *
5	11.14 ± 2.64	72.67 ± 6.80 *	25.14 ± 1.23 *	35.12 ± 1.95 *	53.94 ± 2.14 *	30.67 ± 2.46 *
7	23.43 ± 2.64	82.23 ± 6.70 *	43.79 ± 3.40 *	56.52 ± 2.58 *	71.75 ± 1.95 *	46.53 ± 2.48 *
9	57.85 ± 2.59	94.29 ± 3.37 *	61.65 ± 2.48	68.94 ± 2.22 *	86.46 ± 2.15 *	73.71 ± 2.82 *
12	78.37 ± 2.36	99.83 ± 2.62 *	84.80 ± 2.49 *	98.93 ± 0.39 *	96.84 ± 1.11 *	96.12 ± 1.13 *

Data are expressed as the mean ± SEM (*n* = 5). * *p* < 0.05 indicates a significant difference compared to the negative control (NC) group, as determined by one-way ANOVA followed by Dunnett’s post-hoc test. Animals were assigned to experimental groups through a randomization process using a random number table, and treatments were topically administered daily only during the first three days post-wounding.

**Table 5:** Percentage of wound contraction of the fraction subgroups of the LR-F2 fraction of *B. bipinnata* resin in the skin wound model.

Days	NC	PFD (0.08 g/mL)	Tween 80	LR-F2-A (5.0 mg/mL)	LR-F2-B (5.0 mg/mL)	LR-F2-C (5.0 mg/mL)	LR-F2-D (5.0 mg/mL)	LR-F2-E (5.0 mg/mL)
	(%)							
0	00.00 ± 0.00	00.00 ± 0.00	00.00 ± 0.00	00.00 ± 0.00	00.00 ± 0.00	00.00 ± 0.00	00.00 ± 0.00	00.00 ± 0.00
2	03.59 ± 1.04	59.68 ± 3.59 *	11.63 ± 3.78 *	29.71 ± 3.40 *	11.66 ± 3.10 *	11.92 ± 3.20 *	2.49 ± 2.80 *	18.72 ± 2.60 *
5	11.14 ± 2.64	72.67 ± 6.80 *	25.14 ± 1.23 *	46.53 ± 2.60 *	29.81 ± 2.60 *	19.07 ± 2.10 *	12.68 ± 2.93	32.41 ± 1.40 *
7	23.43 ± 2.64	82.23 ± 6.70 *	43.79 ± 3.40 *	55.62 ± 2.30 *	42.38 ± 2.80 *	21.20 ± 2.70	39.90 ± 2.91 *	50.02 ± 2.01 *
9	57.85 ± 2.59	94.29 ± 3.37 *	61.65 ± 2.48	88.88 ± 3.50	59.06 ± 3.70	31.52 ± 1.90	44.94 ± 1.22 *	77.11 ± 1.09 *
12	78.37 ± 2.36	99.83 ± 2.62	84.80 ± 2.49 *	95.44 ± 3.00	83.74 ± 1.20 *	68.47 ± 1.10	85.33 ± 1.55 *	93.05 ± 3.02

Data are expressed as the mean ± SEM (*n* = 5). * *p* < 0.05 indicates a significant difference compared to the negative control (NC) group, as determined by one-way ANOVA followed by Dunnett’s post-hoc test. Animals were assigned to experimental groups through a randomization process using a random number table, and treatments were topically administered daily only during the first three days post-wounding.

**Table 6:** Percentage of wound contraction caused by the isolated compounds present in the most active fractions of *B. bipinnata* resin in a skin wound model.

Days	NC	Petrolatum	3-Epilupeol (1 μM)	Stigmasterol (1 μM)	Lupenone (1 μM)
	(%)				
0	00.00 ± 0.00	00.00 ± 0.00	00.00 ± 0.00	00.00 ± 0.00	00.00 ± 0.00
2	03.59 ± 1.04	14.12 ± 1.26 *	17.93 ± 0.34 *	26.10 ± 1.48 *	23.50 ± 0.73 *
5	11.14 ± 2.64	27.45 ± 5.05 *	55.49 ± 1.77 *	66.44 ± 3.06 *	47.93 ± 3.19 *
7	23.43 ± 2.64	50.85 ± 3.13 *	60.86 ± 3.13 *	76.13 ± 3.29 *	57.43 ± 1.33 *
9	57.85 ± 2.59	69.15 ± 2.09 *	85.67 ± 3.46 *	95.24 ± 1.22 *	79.52 ± 2.74 *
12	78.37 ± 2.36	85.49 ± 1.63 *	95.74 ± 0.85 *	96.84 ± 1.11 *	92.16 ± 1.47 *

Data are expressed as the mean ± SEM (*n* = 5). * *p* < 0.05 indicates a significant difference compared to the negative control (NC) group, as determined by one-way ANOVA followed by Dunnett’s post-hoc test. Animals were assigned to experimental groups through a randomization process using a random number table, and treatments were topically administered daily only during the first three days post-wounding.

**Table 7:** Semi-quantitative histological assessment of wound healing parameters across experimental groups.

Phase/Day	Histological Parameter	Negative Control	*B. bipinnata* Resin	Stigmasterol (STG)
Day 2	Inflammatory Infiltrate (PMN)	+++	++	+
	Dermal Disorganization	+++	++	+
	Initial Angiogenesis	−	+	+
Day 5–7	Interstitial Edema	+++	++	+
	Epidermal Hyperplasia	−	++	+++
	Fibroblast Proliferation	+	++	+++
Day 9	Granulation Tissue Maturity	+	++	+++
	Hair Follicles (Anagen Phase)	−	+	+++
	Stromal Remodeling	+	++	+++
Day 12	Complete Re-epithelialization	+	++	+++
	Collagen Fiber Organization	+	++	+++
	Adnexal Maturation (Glands)	−	++	+++

Scoring criteria were defined as follows: (−) Absence (no detectable presence); (+) Mild/Scant (minimal features, <25% of the field); (++) Moderate (consistent presence, 25–50% of the field); and (+++) Marked/Complete (extensive presence, >50% of the field). All parameters were evaluated by two independent, blinded observers, and scores represent the consensus of the findings.

**Table 8:** SAR summary of lupane-type triterpenoids at the C-3 position.

Compound	C-3 Configuration	Closure (Day 12, %)	MWHT (Days)
Lupeol acetate (**2**)	3*β-Acetoxy*	N.D.	N.D.
Lupenone (**3**)	3-Oxo (Ketone)	92.16 ± 1.47	10.9 ± 0.6
3-epilupeol (**5**)	3*α-Hydroxyl*	95.74 ± 0.85	10.6 ± 0.5

N.D. = Not Determined in vivo due to yield constraints. Stigmasterol (**1**) is omitted from this table as it belongs to the stigmastane-type steroid class.

## Data Availability

The data presented in this study is available on request from the corresponding author.

## Supplementary Materials

The following supporting information can be downloaded at https://www.mdpi.com/article/10.3390/ph19060931/s1: Table S1. GC-MS Analysis of Compounds Present in *Bursera bipinnata* Resin; Figure S1. Gas Chromatogram of *Bursera bipinnata* Resin Analysis by Gas Chromatography-Mass Spectrometry (GC-MS); Table S2. Spectroscopic and Spectrometric Properties of Stigmasterol; Figure S2. Mass spectrum (EIMS) of stigmasterol (**1**); Figure S3. FT-IR spectrum of stigmasterol (**1**); Figure S4. ^1^H NMR spectrum (400 MHz, CDCl_3_) of stigmasterol (**1**); Figure S5. ^13^C NMR spectrum (400 MHz, CDCl_3_) of stigmasterol (**1**); Table S3. Spectroscopic and Spectrometric Properties of Lupeol acetate; Figure S6. Mass spectrum (EIMS) of lupeol acetate (**2**); Figure S7. FT-IR spectrum of lupeol acetate (**2**); Figure S8. ^1^H NMR spectrum (400 MHz, CDCl_3_) of lupeol acetate (**2**); Figure S9. ^13^C NMR spectrum (400 MHz, CDCl_3_) of lupeol acetate (**2**); Table S4. Spectroscopic and Spectrometric Properties of Lupenone; Figure S10. Mass spectrum (EIMS) of lupenone (**3**); Figure S11. FT-IR spectrum of lupenone (**3**); Figure S12. ^1^H NMR spectrum (400 MHz, CDCl_3_) of lupenone (**3**); Figure S13. ^13^C NMR spectrum (400 MHz, CDCl_3_) of lupenone (**3**); Table S5. Spectroscopic and Spectrometric Properties of Caryophyllene oxide; Figure S14. Mass spectrum (EIMS) of caryophyllene oxide (**4**); Figure S15. FT-IR spectrum of caryophyllene oxide (**4**); Figure S16. ^1^H NMR spectrum (300 MHz, CDCl_3_) of caryophyllene oxide (**4**); Figure S17. ^13^C NMR spectrum (300 MHz, CDCl_3_) of caryophyllene oxide (**4**); Table S6. Spectroscopic and Spectrometric Properties of 3-Epilupeol; Figure S18. Mass spectrum (EIMS) of 3-epilupeol (**5**); Figure S19. FT-IR spectrum of 3-epilupeol (**5**); Figure S20. ^1^H NMR spectrum (300 MHz, CDCl_3_) of 3-epilupeol (**5**); Figure S21. ^13^C NMR spectrum (300 MHz, CDCl_3_) of 3-epilupeol (**5**); Table S7. Purity of isolated compounds; Table S8. Clinical parameters observed in skin wounds after topical application of *B. bipinnata* resin at different doses and treatments (Negative Control, PFD Control, and Vehicle); Table S9. Clinical parameters observed in skin wounds after topical application of the first fractionation groups of *Bursera bipinnata* resin (LR-F1, LR-F2, and LR-F3) and treatments (Negative Control, PFD, and Vehicle); Table S10. Clinical parameters observed in skin wounds after topical application of subfractions (LR-F2-A, LR-F2-B, LR-F2-C, LR-F2-D, and LR-F2-E) and treatments (Negative Control, PFD, and Vehicle); Table S11. Clinical parameters observed in skin wounds after topical application of the compounds: Stigmasterol (**1**), Lupenone (**3**), and 3-Epilupeol (**5**), and Treatments (Negative Control, PFD, and Vehicle).

## Abbreviations

The following abbreviations are used in this manuscript:

^1^H	Proton (hydrogen-1)
^13^C	Carbon-13
ANOVA	Analysis of variance
ATCC	American Type Culture Collection
BALB/c	Bagg albino mice
*Bb*	*Bursera bipinnata*
BCA	Bicinchoninic acid
cAMP	Cyclic adenosine monophosphate
CB2	Cannabinoid receptor type 2
CDCl_3_	Deuterated chloroform
CFU	Colony-forming unit
CIByC	Center for Research in Biodiversity and Conservation
CLSI	Clinical and Laboratory Standards Institute
CONABIO	Comisión Nacional para el Conocimiento y Uso de la Biodiversidad
COX	Cyclooxygenase
DCM	Dichloromethane
DMSO	Dimethyl sulfoxide
ECM	Extracellular matrix
EI	Electron ionization
EIMS	Electron impact mass spectrometry
ELISA	Enzyme-linked immunosorbent assay
FT-IR	Fourier transform infrared spectroscopy
GC–MS	Gas chromatography–mass spectrometry
H&E	Hematoxylin and eosin
IFN	Interferon
IFN-*γ*	Interferon-gamma
IL	Interleukin
IR	Infrared
LPS	Lipopolysaccharide
MCP-1	Monocyte chemoattractant protein-1
MHB	Mueller-Hinton broth
MIC	Minimum inhibitory concentration
MRSA	Methicillin-resistant *Staphylococcus aureus*
MTA	Material transfer agreement
MTT	3-(4,5-dimethylthiazol-2-yl)-2,5-diphenyltetrazolium bromide
MWHT	Mean wound healing time
NC	Negative control
NF	Nuclear factor
NIST	National Institute of Standards and Technology
NMR	Nuclear magnetic resonance
NO	Nitric oxide
NOM	Mexican Official Standards
NSAIDs	Non-steroidal anti-inflammatory drugs
OECD	Organisation for Economic Co-operation and Development
PC	Positive control
PFD	Pirfenidone
PKC	Protein kinase C
PLA2	Phospholipase A2
PMN	Polymorphonuclear leukocytes
SEM	Standard error of the mean
STG	Stigmasterol
TGF-*β*1	Transforming growth factor-beta 1
TLC	Thin-layer chromatography
TLR4	Toll-like receptor 4
TMS	Tetramethylsilane
TNF-*α*	Tumor necrosis factor-alpha
TPA	12-O-tetradecanoylphorbol-13-acetate
UAEM	Universidad Autónoma del Estado de Morelos
UNAM	Universidad Nacional Autónoma de México
UV	Ultraviolet
VC	Vehicle control
VEGF	Vascular endothelial growth factor
