# Supplementary material for: Bioactivity-Guided Isolation of Stigmasterol from Bursera bipinnata Resin: Pharmacological Evidence for Wound-Healing Activity
Source: Pharmaceuticals (Basel). 2026 Jun 12;19(6):931. doi: 10.3390/ph19060931 (PMC13304663; doi:10.3390/ph19060931)
Supplement: Supplementary file 1 [file pharmaceuticals-19-00931-s001.zip › pharmaceuticals-4298010-supplementary.pdf]

4.1. GC-MS Analysis of *Bursera bipinnata* Resin Composition

Table S1. GC-MS Analysis of Compounds Present in *Bursera bipinnata* Resin

Time	Name	Match	Area	Formula	% Area
8.27	o-Cymene	95.3	385170.55	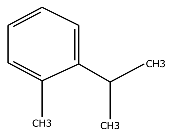	0.11
8.38	D-Limonene	97.7	69100585	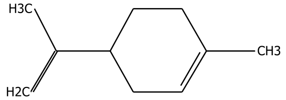	19.15
10.11	trans-p-Mentha-2,8-dienol	95.9	880414.51	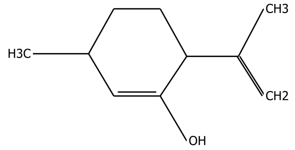	0.24

10.38	cis-p-Mentha-2,8-dien-1-ol	96.6	914954.69	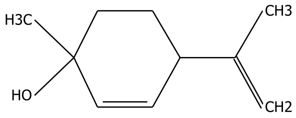	0.25
11.43	.alpha.-Terpineol	98.4	2945844.88	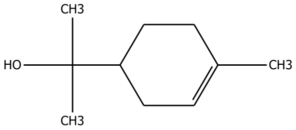	0.82
11.93	2-Cyclohexen-1-ol, 2-methyl-5-(1-methylethenyl)-, cis-	96.6	684750.8	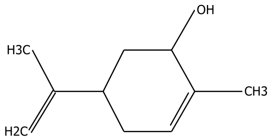	0.19
12.39	D-Carvone	97.7	1121607.92	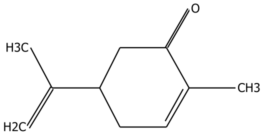	0.31
14.87	(-)-.beta.-Bourbonene	96.9	901975.16	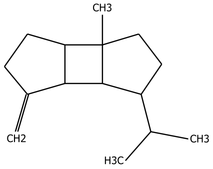	0.25

14.95	Cyclohexane, 1-ethenyl-1-methyl-2,4-bis(1-methylethenyl)-, [1S-(1.alpha.,2.beta.,4.beta.)]-	96.4	1125332.63	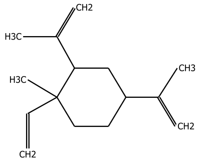	0.31
15.47	Caryophyllene	97.8	34691011.15	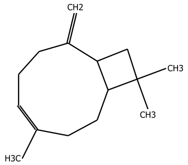	9.61
15.59	.gamma.-Muurolene	92.1	577861.76	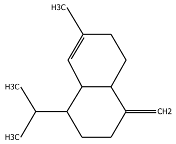	0.16
15.89	.beta.-Panasinsene	94.3	840879.31	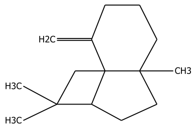	0.23
15.99	1,4,7,-Cycloundecatriene, 1,5,9,9-tetramethyl-, Z,Z,Z-	97.6	1721257.05	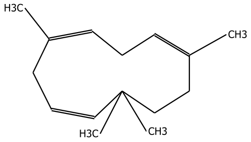	0.48

16.41	(1R,2S,6S,7S,8S)-8-Isopropyl-1-methyl-3-methylenetricyclo[4.4.0.02,7]decane-rel-	97.3	4613698.3	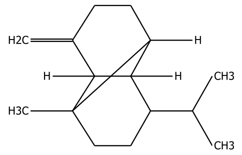	1.28
17.16	Seychellene	88.2	471100.94	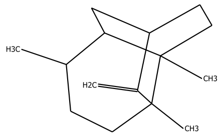	0.13
17.51	Isoaromadendrene epoxide	90.8	1081127.07	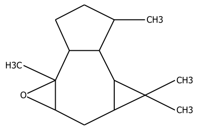	0.30
17.83	Diepicedrene-1-oxide	89.5	476494.14	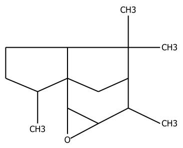	0.13
18.00	Caryophyllene oxide	95.9	29711318.78	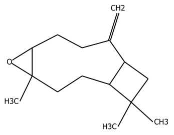	8.23
18.36	(1R,3E,7E,11R)-1,5,5,8-Tetramethyl-12-oxabicyclo[9.1.0]dodeca-3,7-diene	95.1	991142.51	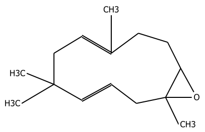	0.27

33.99	Not Identified	-	1799401.28		0.50
34.59	Olean-12-en-3-ol, acetate, (3.beta.)-	85.6	491934.67	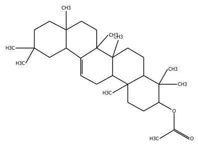	0.14
34.83	Not Identified	-	845570.29		0.23
36.17	Not Identified	-	17766483.58		4.92
36.51	Not Identified	-	18296399.43		5.07
36.68	Lupeol	86.6	70731175.89	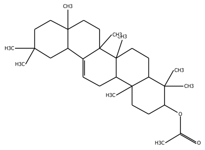	19.60
36.83	Olean-12-en-3-ol, acetate, (3.beta.)-	88.1	20260346.79	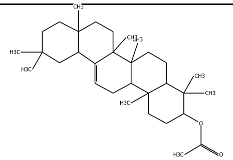	5.61
36.92	Lup-20(29)-en-3-ol, acetate, (3.beta.)-	89.0	64965969.55	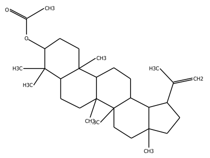	18.00

37.24	Not Identifie	-	5510940.84		1.53
37.59	Not Identified	-	638093.9		0.18
37.94	12-Oleanen-3-yl acetate, (3.alpha.)-	88.2	3965030.81	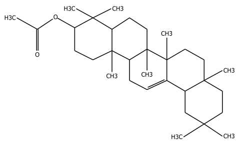	1.10
38.92	No identificado	-	2391596.91		0.66
		**Total Area**	360899471.1		

This table provides the results of the GC-MS analysis of compounds isolated from Bursera bipinnata resin. The identified compounds, their retention times (RT), and molecular weights (MW) are listed. The table also includes the relative peak intensities (%) of each compound, highlighting the most abundant bioactive components detected in the resin. This data is essential for understanding the chemical composition and potential bioactivity of the resin

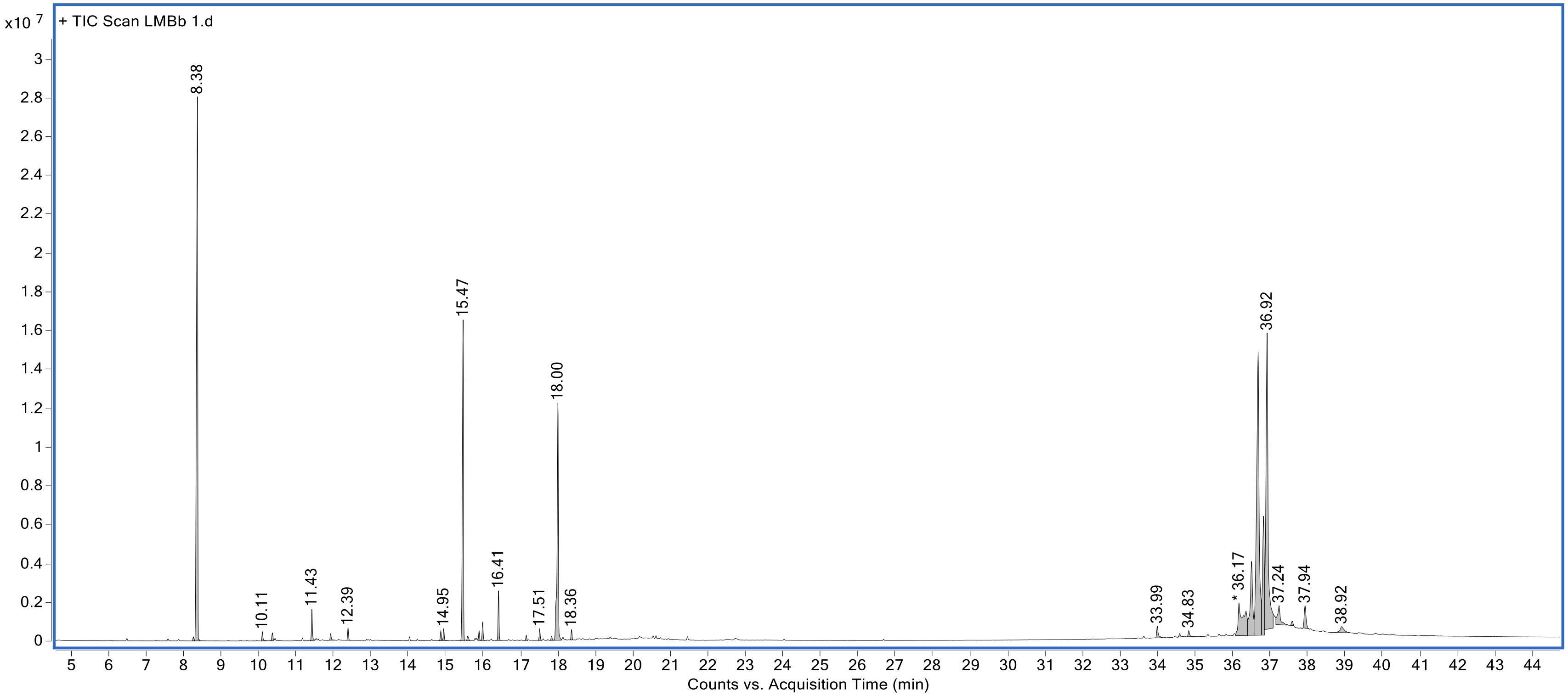

Figure S1. Gas Chromatogram of *Bursera bipinnata* Resin Analysis by Gas Chromatography-Mass Spectrometry (GC-MS). This figure presents the gas chromatogram obtained from the GC-MS analysis of *Bursera bipinnata* resin. The chromatogram shows the separation of individual compounds present in the resin, with each peak corresponding to a specific compound based on its retention time. The identified compounds, as listed in Table 1, were detected in the resin, and their relative abundances are represented by the peak intensities. This analysis provides a comprehensive overview of the chemical composition of the resin, which is essential for understanding its potential bioactive properties.

4.1. Supplementary Spectroscopic Analysis: IR, EIMS, and NMR of Compounds Isolated from the Fraction with Best Wound Healing Activity of *Bursera bipinnata* Resin

This section provides supplementary spectroscopic data supporting the identification and characterization of the compounds isolated from the most active fraction of *B. bipinnata* resin. The analysis includes Infrared (IR) spectroscopy, Electron Ionization Mass Spectrometry (EIMS), and Nuclear Magnetic Resonance (NMR) spectroscopy.

Table S2. Spectroscopic and Spectrometric Properties of Stigmasterol

STRUCTURE

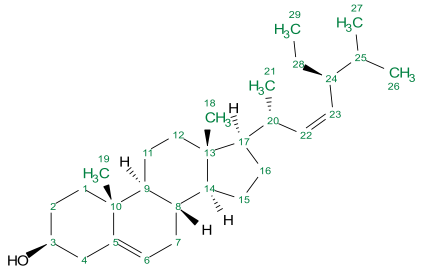

**NAME**	Stigmasterol							
**TECHNIQUE**	NMR				EIMS		IR	
**SOLVENT**	$CDCl_3$				(m/z)	Assignment	(cm^{-1})	Assignment
**FREQUENCY**	400 MHz				412	[$C_{29}H_{48}O$, M+] (3)	3488	Alcohol
**TECHNIQUE**	^{13}C NMR		^{1}H NMR		397	[$M-CH_3]^+$ (3)	3073	Olefinic
**CHEMICAL SHIFTS**	δ_C (ppm)	Assignment	δ_H (ppm)	Multiplicity (*J* in Hz)	368	[$M-C_2H_3]^+$ (2)	2943	Aliphatic
	38.0	C-1	1.84	m	339	[$M-C_2H_5$]+ (3)	2867	Aliphatic
	30.2	C-2	1.50	m	295	[$M-CH_2$]+ (1)	1641	Alkene
	71.8	C-3	3.52	m	257	[M-O]+ (3)	1454	Methylene/methyl
	38.3	C-4	2.27	m	203	[$M-CH_3$]+ (5)	1380	Methyl
	140.7	C-5	-	-	177	[$M-C_2H_2$]+ (4)		
	122.7	C-6	5.35	m	123	[$M-CH_2$]+ (15)		
	32.0	C-7	1.98	m	97	[$M-C_2H_2$]+ (33)		
	31.9	C-8	1.45	m	85	[M-C]+ (3) (45)		
	49.5	C-9	0.95	m	71	[$M-CH_2$]+ (62)		

	35.4	C-10	-	-	57	[M-CH2$_2$]+ (100)
	26.5	C-11	1.50	m	43	[M-CH_2]+ (59)
	40.1	C-12	1.15	m		
	40.5	C-13	-	-		
	56.8	C-14	1.05	m		
	27.5	C-15	1.55	m		
	29.9	C-16	1.25	m		
	56.0	C-17	1.10	m		
	12.2	C-18	0.70	s		
	19.4	C-19	1.01	s		
	40.5	C-20	1.55	m		
	21.2	C-21	0.92	d		
	135.7	C-22	5.15	dd		
	129.3	C-23	5.01	dd		
	53.7	C-24	1.52	m		
	30.0	C-25	1.65	m		
	22.8	C-26	0.84	d		
	22.2	C-27	0.82	d		
	25.4	C-28	1.48	m		
	12.0	C-29	0.80	t		

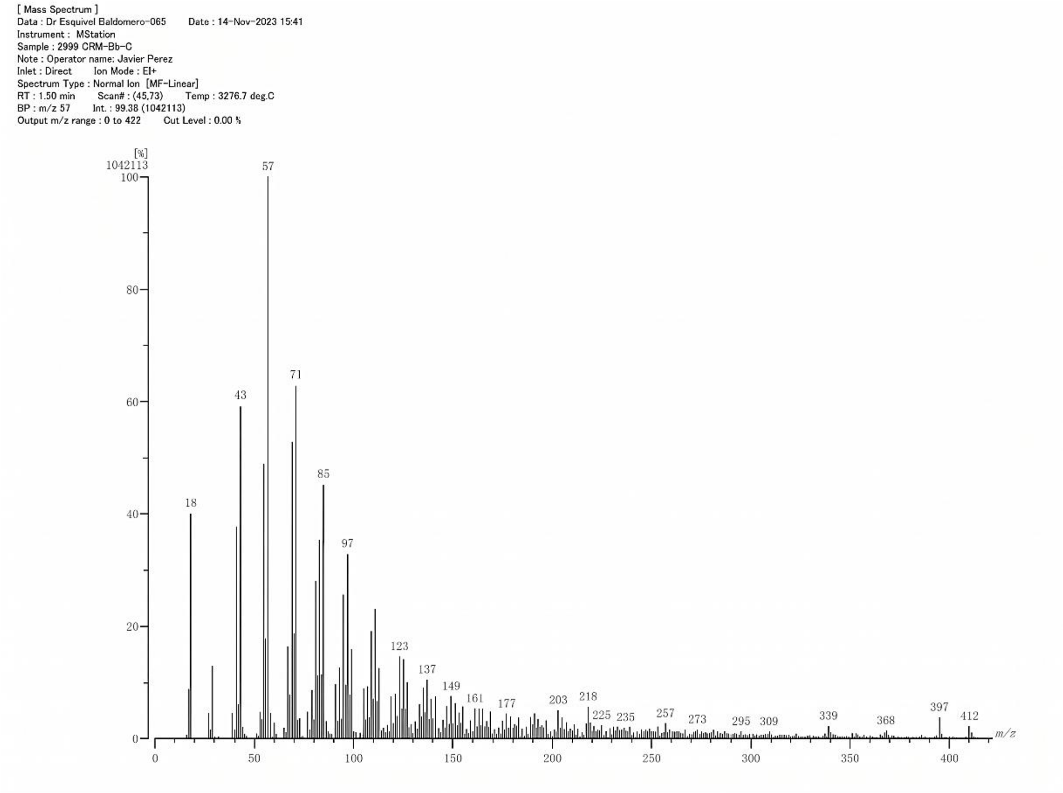

Figure S2. Mass spectrum (EIMS) of stigmasterol (**1**).

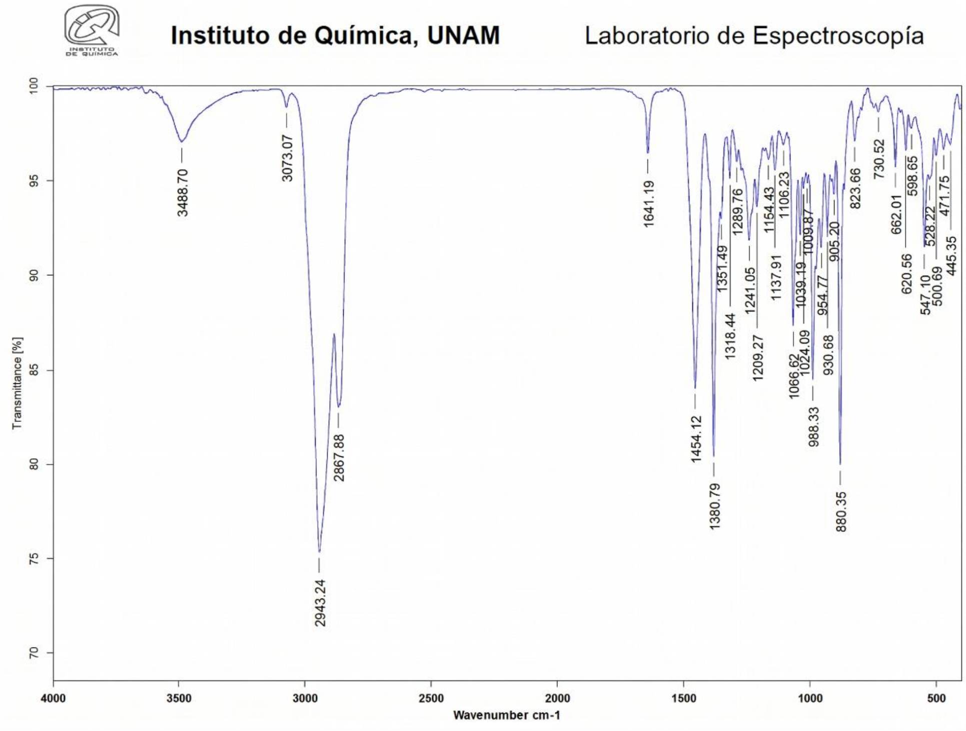

Figure S3. FT-IR spectrum of stigmasterol (**1**).

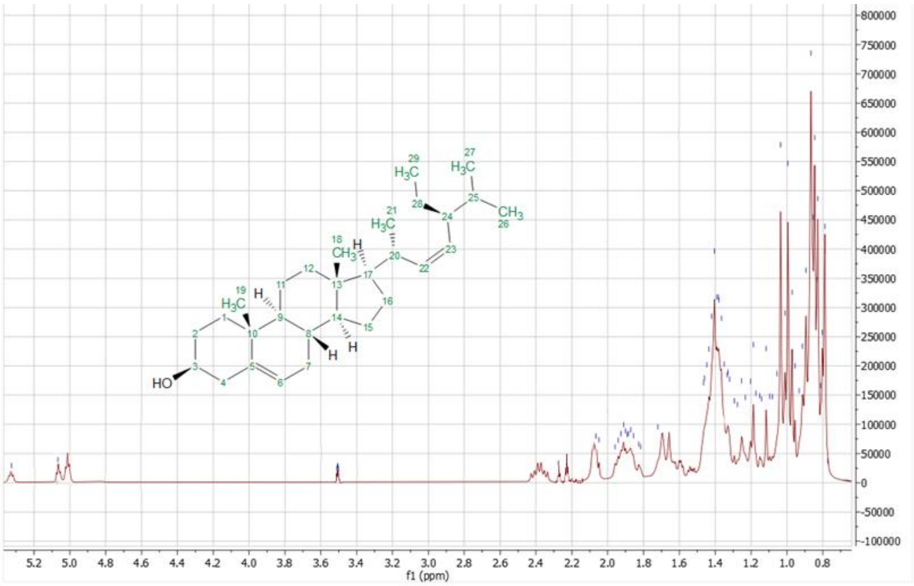

Figure S4. ^{1}H NMR spectrum (400 MHz, $CDCl_3$) of stigmasterol (**1**).

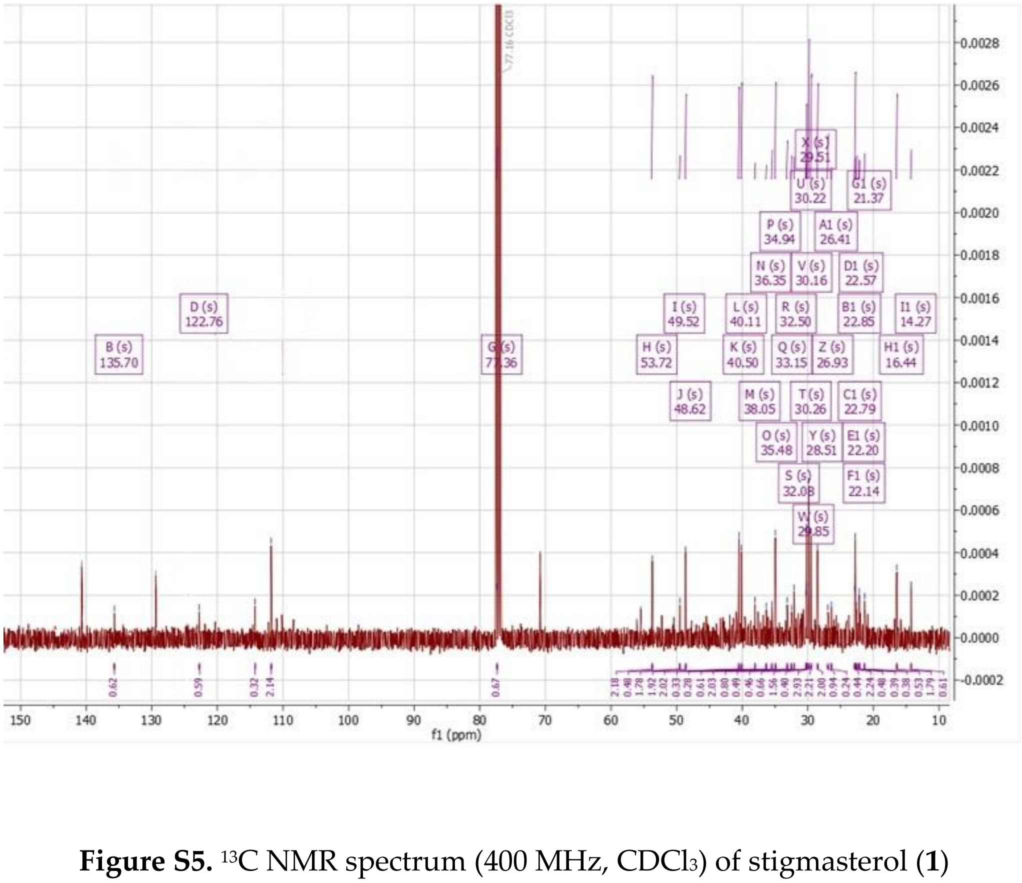

Figure S5. ^{13}C NMR spectrum (400 MHz, $CDCl_3$) of stigmasterol (**1**)

Table S3. Spectroscopic and Spectrometric Properties of Lupeol acetate

STRUCTURE

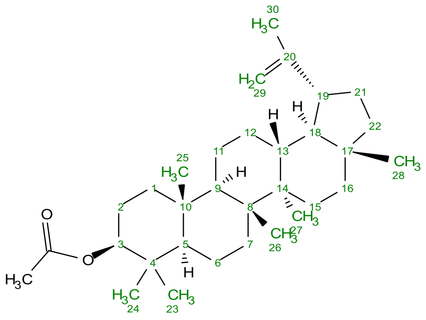

**NAME**	Lupeol acetate							
**TECHNIQUE**	NMR				EIMS		IR	
**SOLVENT**	$CDCl_3$				(m/z)	Assignment	(cm^{-1})	Assignment
**FREQUENCY**	400 MHz				468	[$C_{32}H_{52}O_2$, M+] (12)	3070	Alkene
**TECHNIQUE**	^{13}C NMR		^{1}H NMR		453	$[M-CH_3]^+$ (4)	2942	Aliphatic
**CHEMICAL SHIFTS**	δ_C (ppm)	Assignment	δ_H (ppm)	Multiplicity (J in Hz)	408	[M-CH3COOH]$^+$ (5)	1735	Ester group
	38.1	C-1	1.40	m	393	$[M-CH_3]^+$ (8)	1640	Alkene
	23.0	C-2	1.60	m	249	$[M-CH_3]^+$ (6)	881	Exomethylene
	78.5	C-3	4.47	dd	218	$[M-C_{16}H_{26}O_2]^+$ (100)		
	36.8	C-4	-	-	189	$[(M-C_{16}H_{27})$ - $AcOH]^+$ (45)		
	59.2	C-5	0.85	m	175	$[M-CH_2]^+$ (15)		
	18.2	C-6	1.45	m	121	$[(M-C_{21}H_{35})$ - $AcOH]^+$ (22)		
	34.0	C-7	1.40	m				
	41.1	C-8	-	-				
	50.3	C-9	1.25	m				
	35.7	C-10	-	-				
	21.5	C-11	1.40	m				
	25.2	C-12	1.05	m				
	37.3	C-13	1.65	m				
	43.0	C-14	-	-				

	27.6	C-15	1.00	m
	34.2	C-16	1.50	m
	43.1	C-17	-	-
	48.1	C-18	1.35	m
	48.4	C-19	2.38	m
	151.1	C-20	-	-
	30.0	C-21	1.90	m
	40.1	C-22	1.20	m
	27.9	C-23	0.86	s
	16.4	C-24	0.83	s
	16.0	C-25	0.84	s
	16.0	C-26	1.00	s
	14.52	C-27	0.98	s
	18.1	C-28	0.79	s
	20.9	C-29	4.69, 4.57	s
	109.4	C-30	1.68	s
	171.1	CH_3COO	-	-
	21.32	CH_3COO	2.04	s

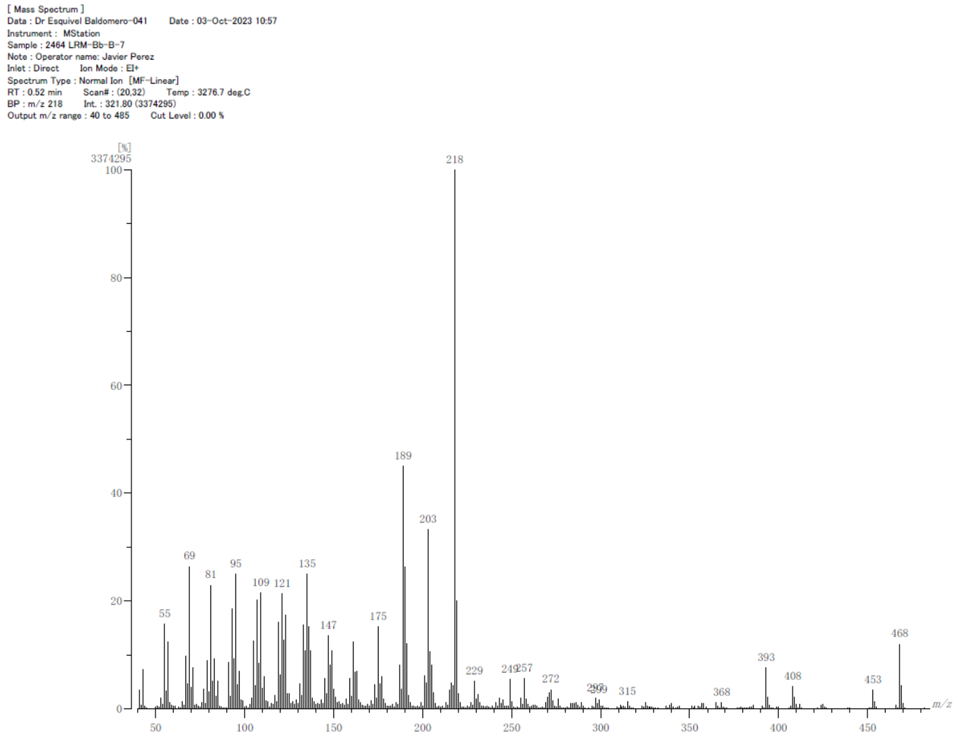

Figure S6. Mass spectrum (EIMS) of lupeol acetate (**2**).

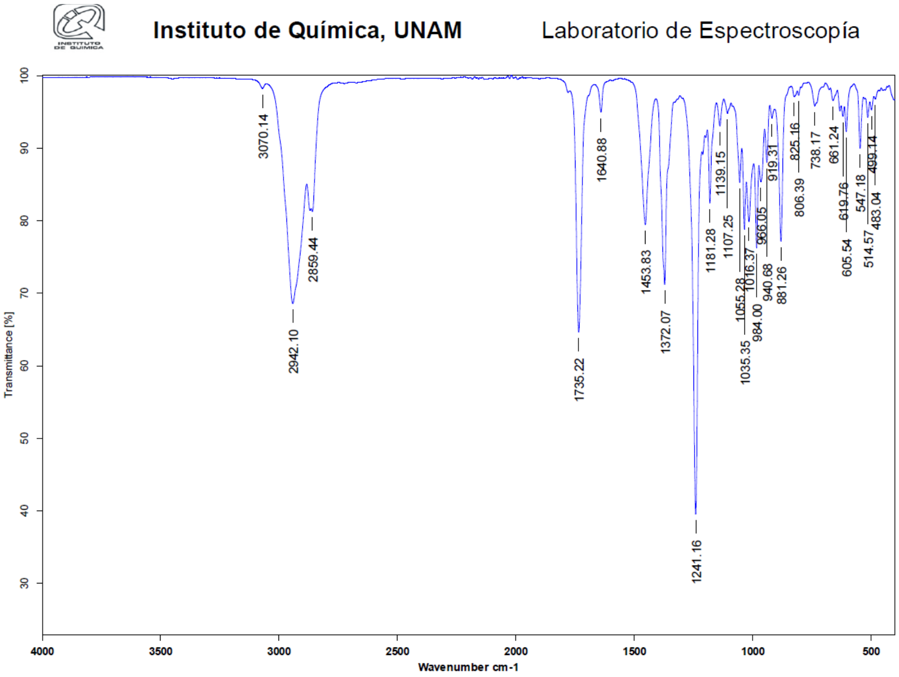

Figure S7. FT-IR spectrum of lupeol acetate (**2**).

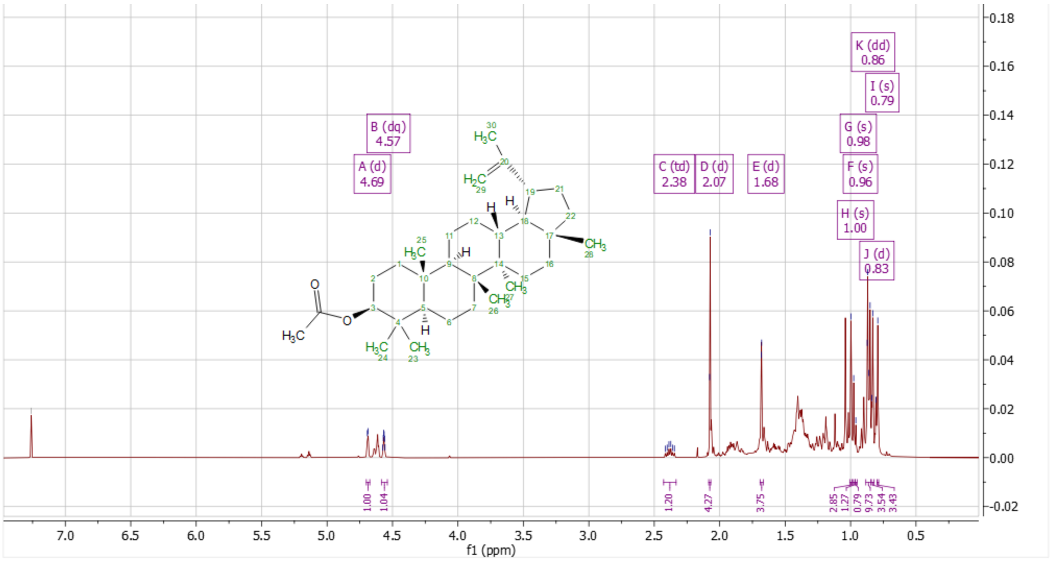

Figure S8. ^{1}H NMR spectrum (400 MHz, $CDCl_3$) of lupeol acetate (**2**).

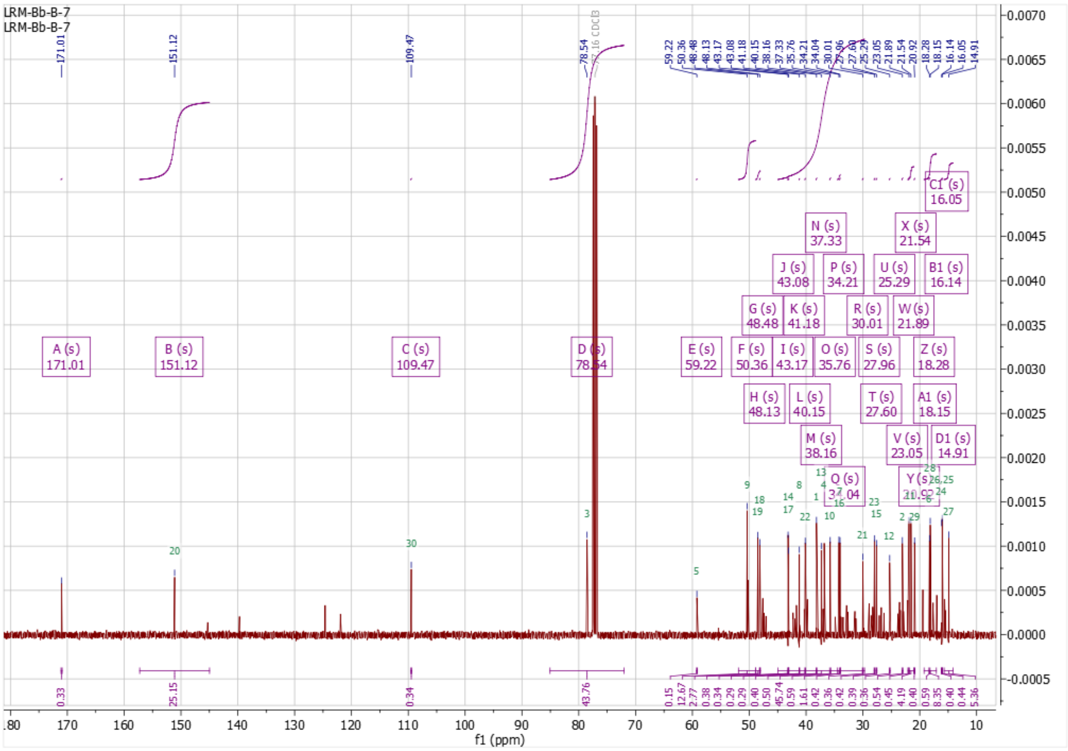

Figure S9. ^{13}C NMR spectrum (400 MHz, $CDCl_3$) of lupeol acetate (**2**)

Table S4. Spectroscopic and Spectrometric Properties of Lupenone

STRUCTURE

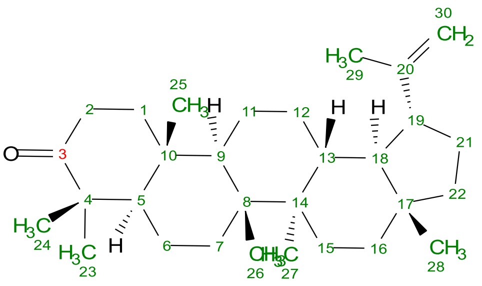

NAME	Lupenone							
TECHNIQUE	NMR				EIMS		IR	
SOLVENT	$CDCl_3$				(m/z)	Assignment	(cm^{-1})	Assignment
FREQUENCY	400 MHz				424	[$C_{30}H_{48}O$, M+] (31)	3070	Alkene
TECHNIQUE	^{13}C NMR		^{1}H NMR		409	[M-CH_3]+ (18)	2943	Aliphatic
CHEMICAL SHIFTS	δC (ppm)	Assignment	δH (ppm)	Multiplicity (J in Hz)	381	[M-CH_2]+ (5)	1705	Ketone
	38.3	C-1	1.90	m	355	[M-CH]+ (4)	1640	Alkene
	34.3	C-2	2.43	m	313	[M-C2H_5]+ (11)	1380	Methyl groups
	218.0	C-3	-	-	285	[M-CH_3]+ (3)		
	47.4	C-4	-	-	257	[M-CH_2]+ (6)		
	55.0	C-5	1.30	m	245	[M-C]+ (12)		
	19.8	C-6	1.45	m	218	[M-CH_2]+ (90)		
	33.7	C-7	1.40	m	205	[M-CH]+ (48)		
	40.9	C-8	-	-	135	[M-C]+ (43)		
	49.9	C-9	1.25	m	109	[M-CH_2]+ (61)		
	37.0	C-10	-	-	57	[M-C_2]+ (100)		
	21.1	C-11	1.40	m				
	25.3	C-12	1.05	m				
	39.7	C-13	1.65	m				
	43.0	C-14	-	-				
	27.5	C-15	1.00	m				

	35.6	C-16	1.50	m
	43.1	C-17	-	-
	48.4	C-18	1.35	m
	48.1	C-19	2..380	m
	151.0	C-20	-	-
	29.9	C-21	1.90	m
	40.1	C-22	1.20	m
	26.7	C-23	1.05	d
	41.6	C-24	1.01	d
	15.9	C-25	0.98	s
	16.1	C-26	0.93	dd
	14.6	C-27	0.78	s
	18.1	C-28	-	-
	109.5	C-29	4.68, 4.56	d, dd
	19.8	C-30	1.67	m

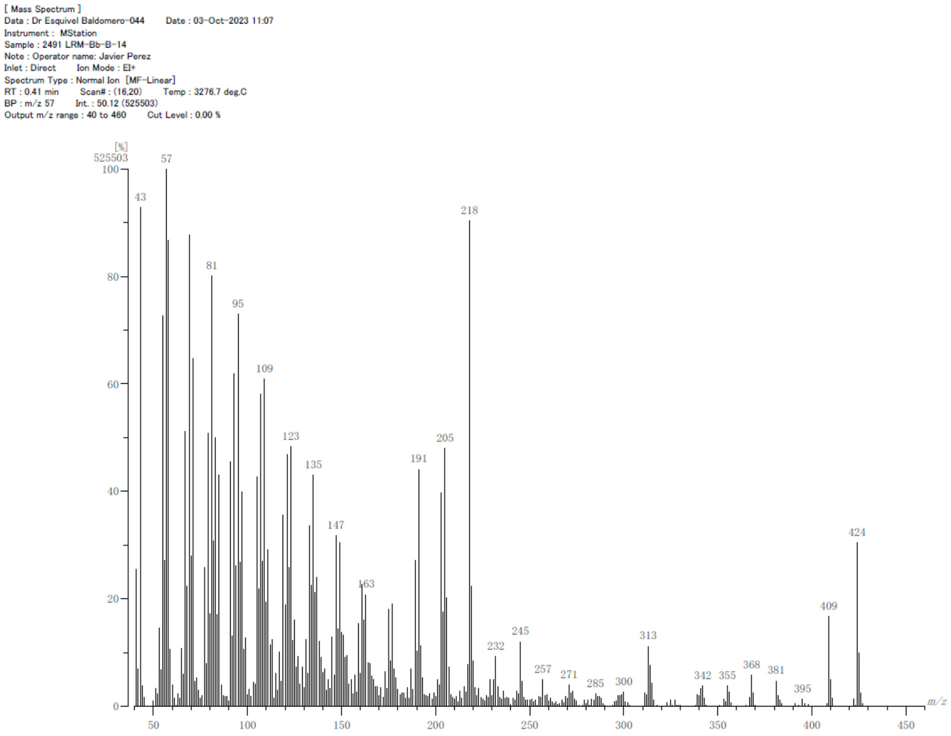

Figure S10. Mass spectrum (EIMS) of lupenone (**3**).

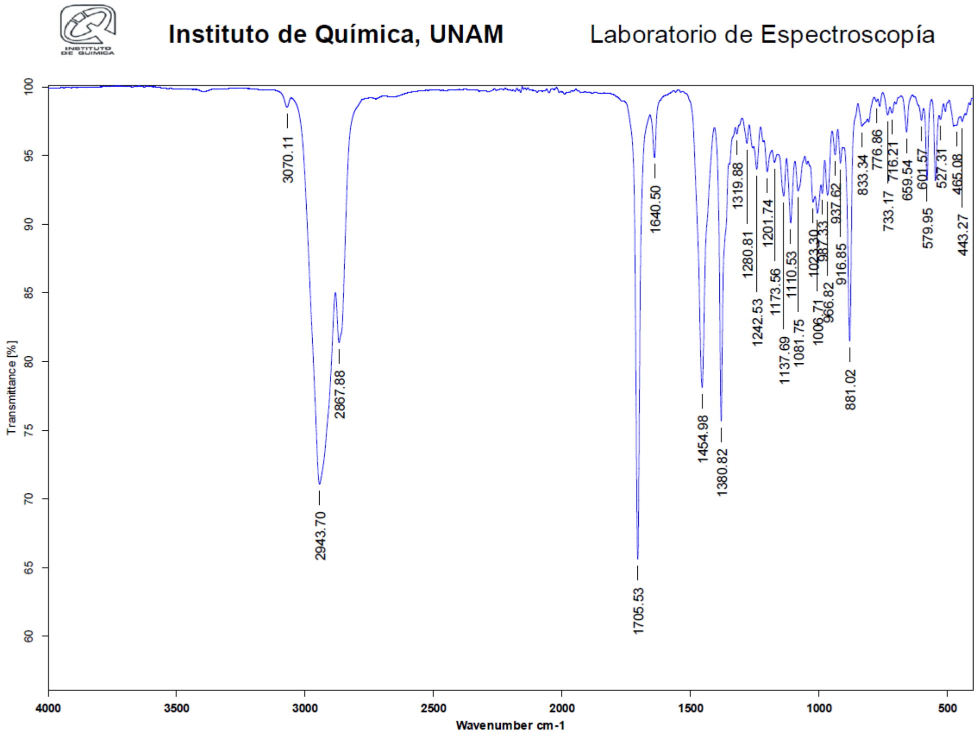

Figure S11. FT-IR spectrum of lupenone (**3**).

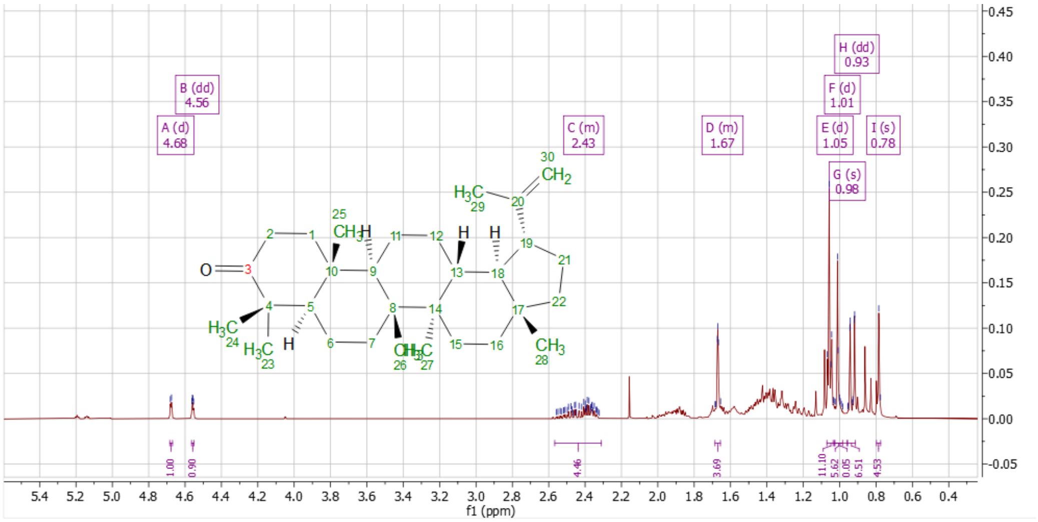

Figure S12. ^{1}H NMR spectrum (400 MHz, $CDCl_3$) of lupenone (**3**).

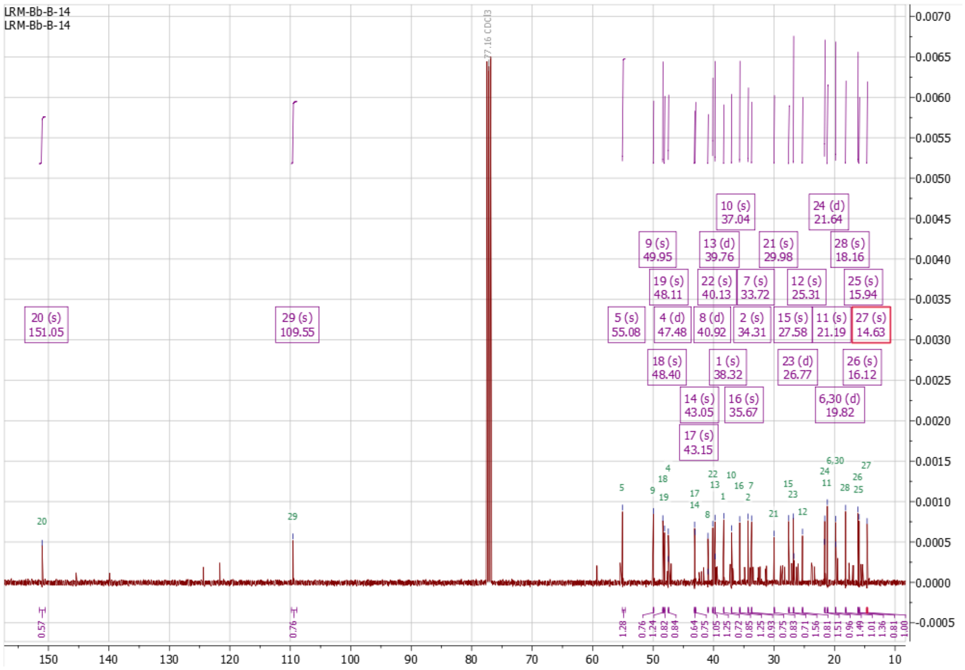

Figure S13. ^{13}C NMR spectrum (400 MHz, $CDCl_3$) of lupenone (**3**).

Table S5. Spectroscopic and Spectrometric Properties of Caryophyllene oxide

**STRUCTURE**	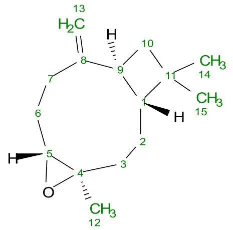							
**NAME**	Caryophyllene oxide							
**TECHNIQUE**	NMR				EIMS		IR	
**SOLVENT**	$CDCl_3$				(m/z)	Assignment	(cm^{-1})	Assignment
**FREQUENCY**	300 MHz				220	[$C_{15}H_{24}O$, M+] (4)	3067	Alkene
**TECHNIQUE**	^{13}C NMR		^{1}H NMR		205	[M-CH_3] + (7)	1630	Alkene
**CHEMICAL SHIFTS**	δ_C (ppm)	Assignment	δH (ppm)	Multiplicity (J in Hz)	187	[M-H_2O] + (7)	862	Exomethylene
	50.8	C-1	1.75	m	163	[M-CH_2] + (11)		
	27.3	C-2	1.45	m	106	[M-CH_3] + (67)		
	39.3	C-3	2.20	m	93	[M-CH] + (100)		
	63.9	C-4	-	-	79	[M-CH_2] + (100)		
	63.9	C-5	2.88	dd	55	[M-CH_2] + (45)		
	30.0	C-6	1.30	m				
	29.9	C-7	1.55	m				
	152.0	C-8	-	-				
	48.9	C-9	2.61	m				
	39.9	C-10	1.60	m				
	34.2	C-11	-	-				
	17.1	C-12	1.24	s				
	112.9	C-13	4.85, 4.97	s				
	21.8	C-14	0.98	s				
	30.3	C-15	1.01	s				

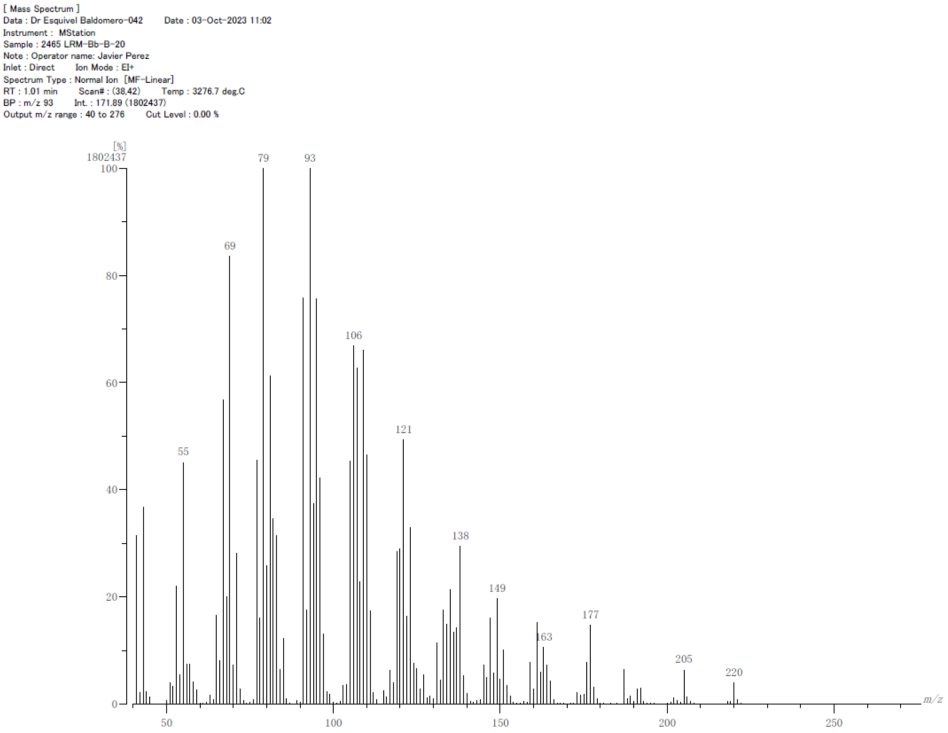

Figure S14. Mass spectrum (EIMS) of caryophyllene oxide (**4**).

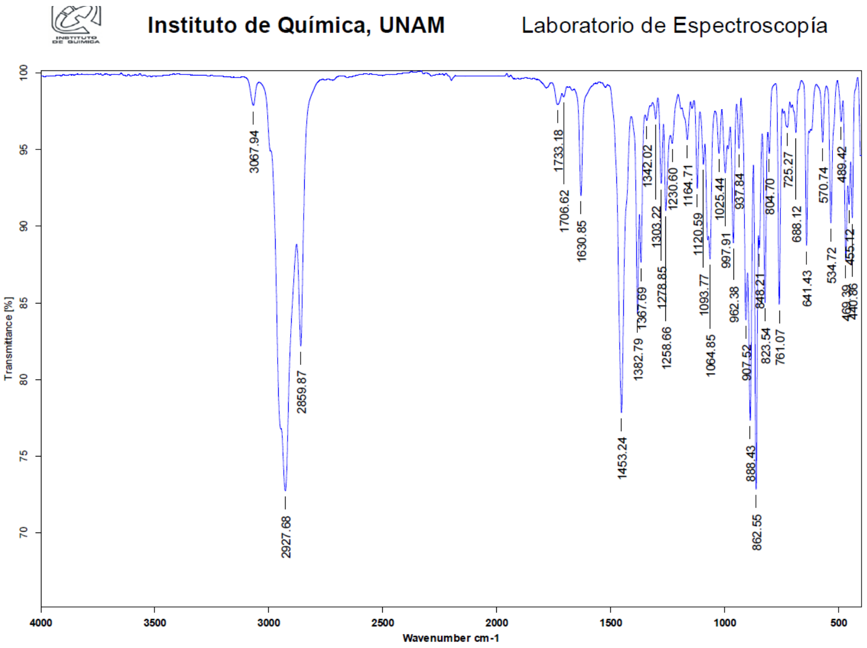

Figure S15. FT-IR spectrum of caryophyllene oxide (**4**).

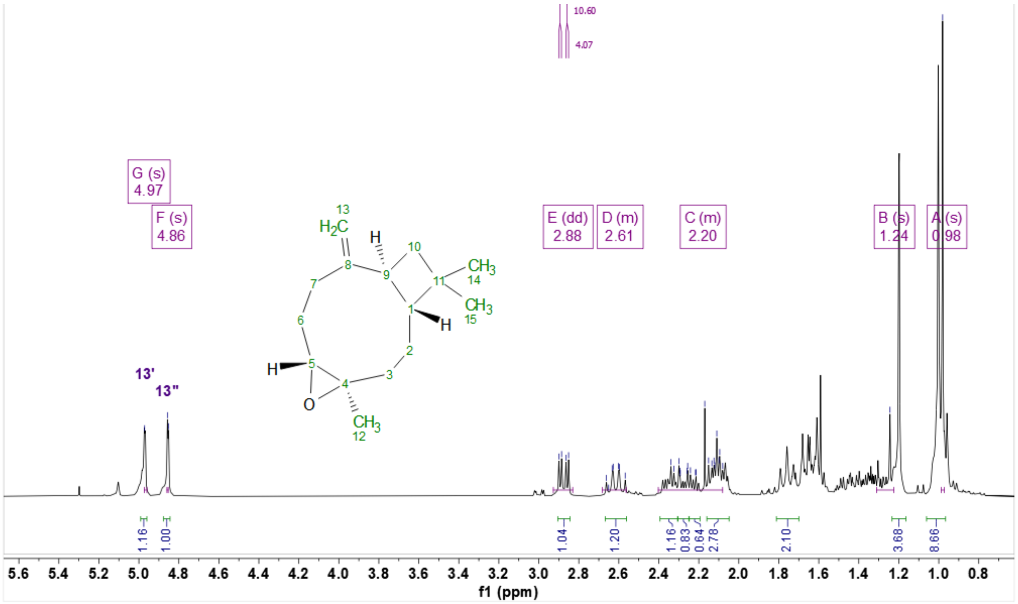

Figure S16. ^{1}H NMR spectrum (300 MHz, $CDCl_3$) of caryophyllene oxide (**4**).

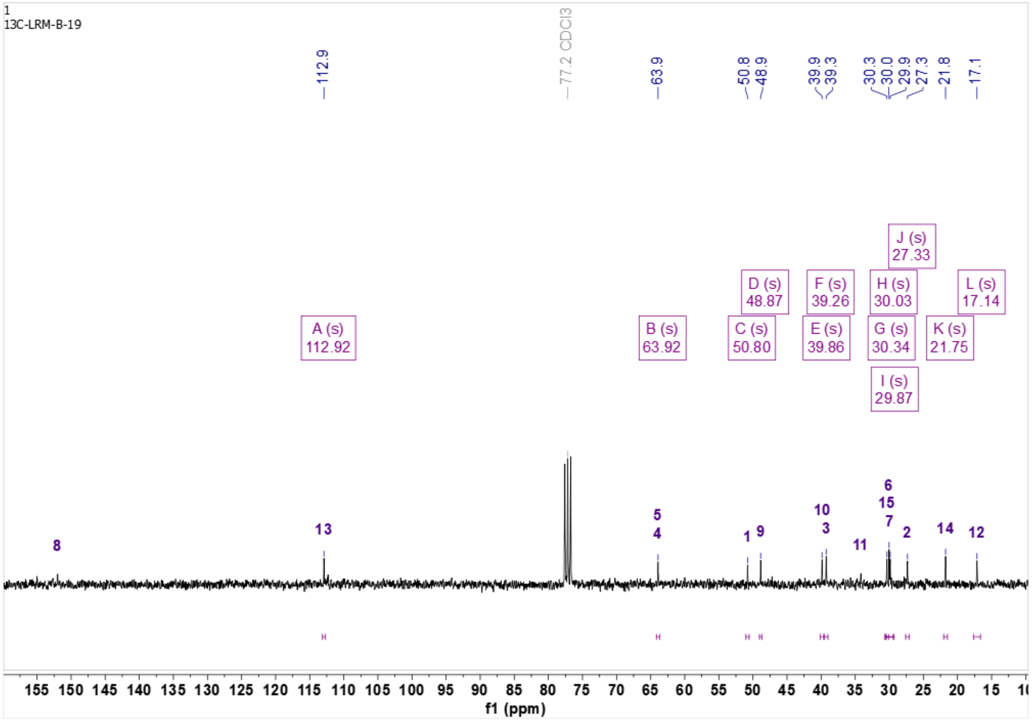

Figure S17. ^{13}C NMR spectrum (300 MHz, $CDCl_3$) of caryophyllene oxide (**4**).

Table S6. Spectroscopic and Spectrometric Properties of 3-Epilupeol

STRUCTURE

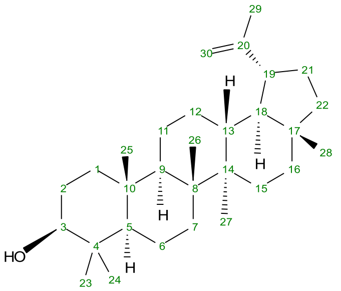

NAME	3-Epilupeol							
TECHNIQUE	NMR				EIMS		IR	
SOLVENT	$CDCl_3$				(m/z)	Assignment	(cm^{-1})	Assignment
FREQUENCY	300 MHz				426	[$C_{30}H_{50}O$, M+] (50)	3484	Alcohol
TECHNIQUE	^{13}C NMR		^{1}H NMR		411	[M-CH_3]+ (15)	3072	Alkene
CHEMICAL SHIFTS	δC (ppm)	Assignment	δH (ppm)	Multiplicity (J in Hz)	393	[M-H_2O]+ (18)	1641	Alkene
	37.6	C-1	1.42	m	257	[M-CH_3]+ (19)	879	Exomethylene
	27.5	C-2	1.60	m	218	[M-$C_{14}H_{24}O$]+ (100)		
	76.9	C-3	3.38	q	203	[M-CH_3]+ (56)		
	38.1	C-4	-	-	189	[M-$C_{16}H_{29}O$]+ (60)		
	50.3	C-5	1.30	m	135	[M-$C_{21}H_{39}$]+ (51)		
	19.4	C-6	1.45	m				
	33.35	C-7	1.40	m				
	48.4	C-8	-	-				
	49.12	C-9	1.25	m				
	35.7	C-10	-	-				
	22.3	C-11	1.40	m				
	25.2	C-12	1.05	m				

	37.4	C-13	1.65	m
	41.1	C-14	-	-
	25.5	C-15	1.00	m
	34.2	C-16	1.50	m
	43.0	C-17	-	-
	48.3	C-18	1.35	m
	43.1	C-19	2.38	td
	151.2	C-20	-	-
	29.9	C-21	1.90	m
	40.1	C-22	1.20	m
	28.4	C-23	1.03	s
	16.0	C-24	0.96	s
	18.1	C-25	0.93	s
	16.1	C-26	0.82	s
	14.7	C-27	0.78	s
	18.4	C-28	-	-
	20.9	C-29	1.68	s
	109.5	C-30	4.68, 4.56	d, dt

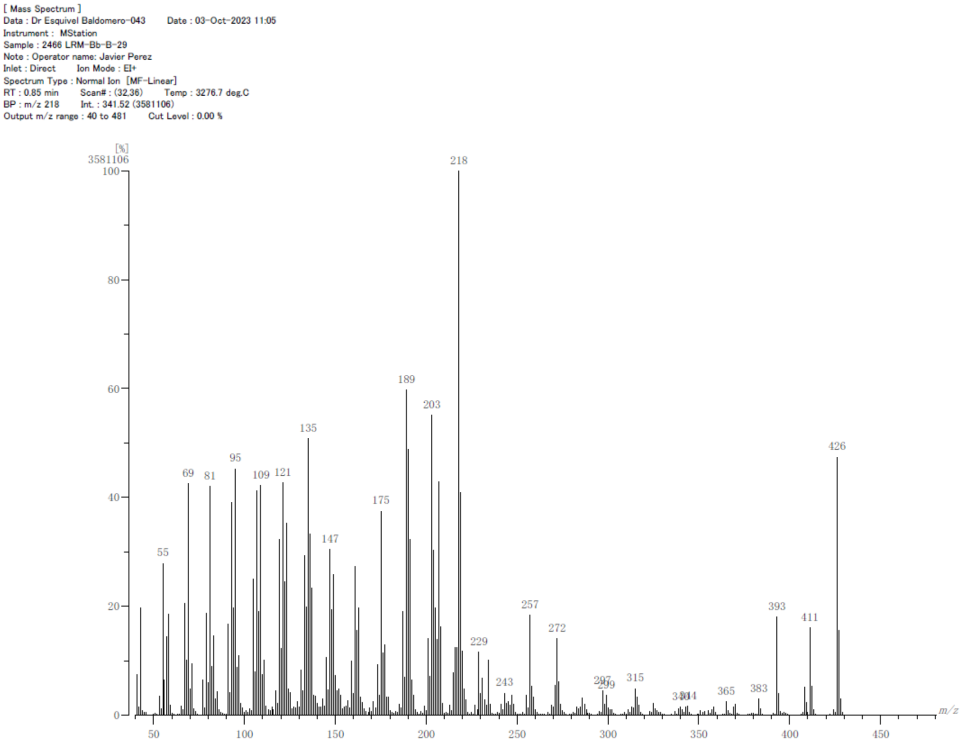

Figure S18. Mass spectrum (EIMS) of 3-epilupeol (**5**).

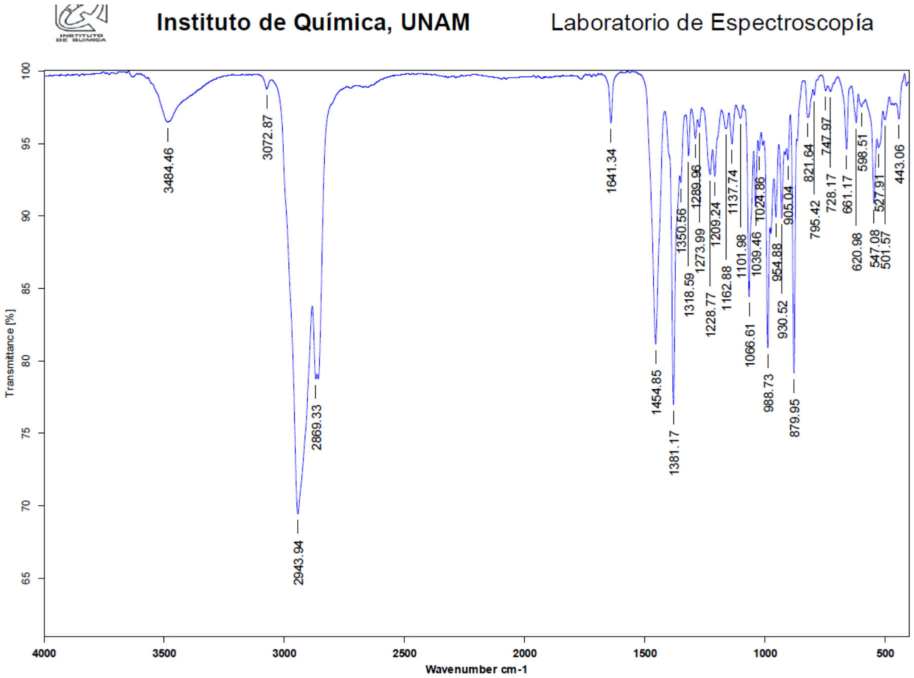

Figure S19. FT-IR spectrum of 3-epilupeol (**5**)

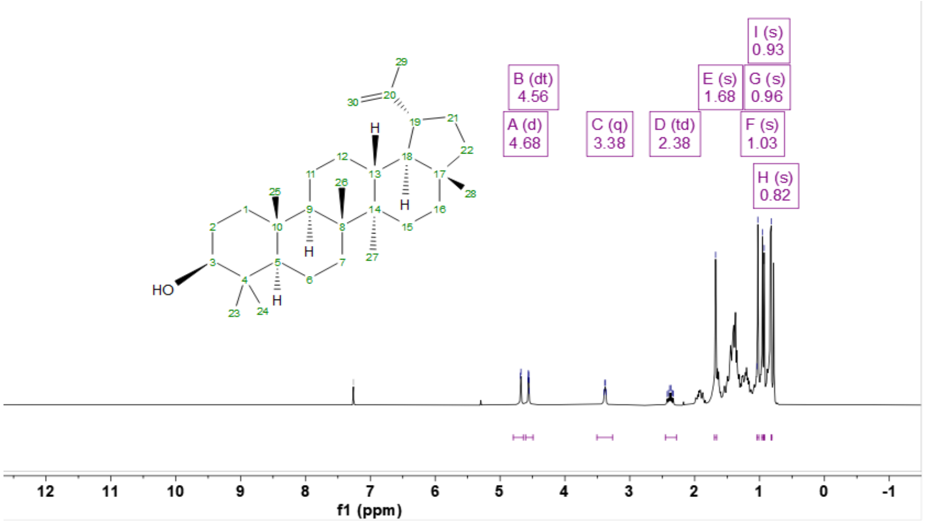

Figure S20. ^1H NMR spectrum (300 MHz, $CDCl_3$) of 3-epilupeol (**5**).

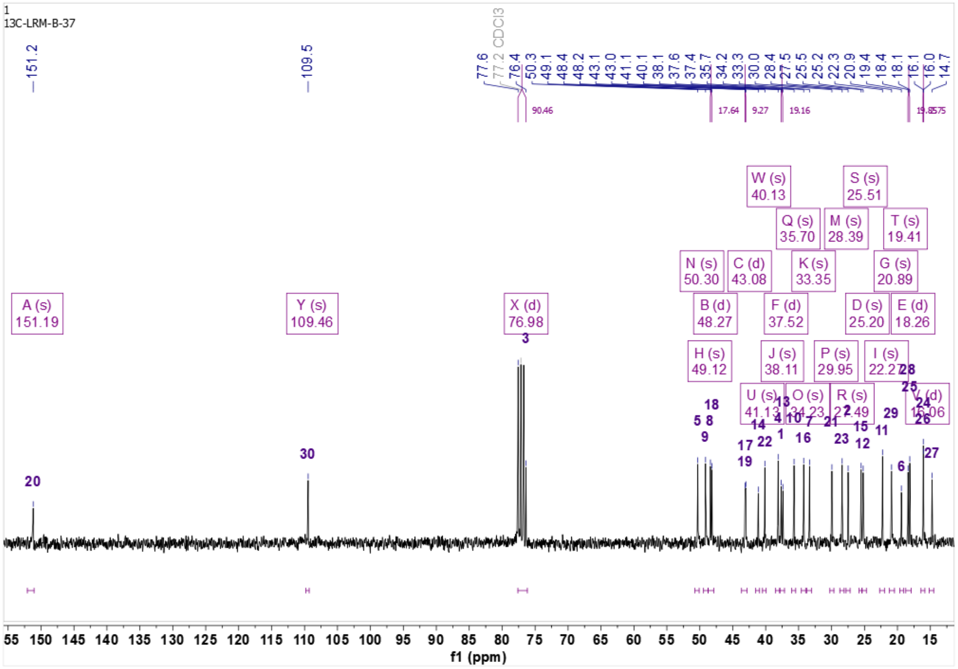

Figure S21. ^{13}C NMR spectrum (300 MHz, $CDCl_3$) of 3-epilupeol (**5**).

Table S7. Purity and testing status of isolated compounds.

Compound	Identification	Purity Range*	Biologically tested
1	Stigmasterol	95 %	Yes
2	Lupeol acetate	92 %	No
3	Lupenone	97 %	Yes
4	Caryophyllene oxide	96 %	No
5	3-Epilupeol	95 %	Yes

* Purity was estimated by GC-MS area normalization (Match factors > 90%) and NMR baseline analysis. The range accounts for instrumental noise and minor co-eluting isomeric traces typical of

4.3. Evaluation of Macroscopic Wound Characteristics in the Mouse Skin Wound Model

Table S8. Clinical parameters observed in skin wounds after topical application of *B. bipinnata* resin at different doses and treatments (Negative Control, PFD Control, and Vehicle).

Parameters	Treatments	Days after wound induction			
		Day 2	Day 7	Day 12	Day 14
Exudate	NC	1	-	-	-
	PFD 0.08 g	1	-	-	-
	Tween 80	-	-	-	-
	Bb resin 0.12 g/mL	1	-	-	-
	Bb resin 0.25 g/mL	-	-	-	-
	Bb resin 0.50 g/mL	1	-	-	-
Edema	NC	3	2	1	1
	PFD 0.08 g	1	-	-	-
	Tween 80	2	1	-	-
	Bb resin 0.12 g/mL	1	2	-	-
	Bb resin 0.25 g/mL	1	1	-	-
	Bb resin 0.50 g/mL	-	-	-	-
Hemorrhage	NC	-	-	-	-
	PFD 0.08 g	-	-	-	-
	Tween 80	-	-	-	-
	Bb resin 0.12 g/mL	-	-	-	-
	Bb resin 0.25 g/mL	-	-	-	-
	Bb resin 0.50 g/mL	-	-	-	-
Crust	NC	1	3	3	2
	PFD 0.08 g	3	5	1	-
	Tween 80	1	3	3	2
	Bb resin 0.12 g/mL	2	3	2	-
	Bb resin 0.25 g/mL	2	4	2	-
	Bb resin 0.50 g/mL	3	5	2	-
Granulation	NC	-	-	-	1
	PFD 0.08 g	-	-	2	5
	Tween 80	-	-	-	2
	Bb resin 0.12 g/mL	-	-	1	4
	Bb resin 0.25 g/mL	-	-	1	5
	Bb resin 0.50 g/mL	-	-	1	5

This table summarizes the clinical parameters observed in skin wounds following the topical application of *B. bipinnata* resin at various concentrations (0.12 g/mL, 0.25 g/mL, and 0.50 g/mL). The clinical parameters include wound closure percentages, signs of inflammation, edema, scab formation, and any other relevant observations recorded at different time points (Days 2, 7, 12, and 14). Comparisons were made between the resin treatments vs the negative control (NC). The sample size for each treatment group was $n = 5$.

Table S9. Clinical parameters observed in skin wounds after topical application of the first fractionation groups of *Bursera bipinnata* resin (LR-F1, LR-F2, and LR-F3) and treatments (Negative Control, PFD, and Vehicle).

Parameters	Treatments	Days after wound induction			
		Day 2	Day 7	Day 12	Day 14
Exudate	NC	2	-	-	-
	PFD 0.08 g/mL	2	-	-	-
	Tween 80	-	-	-	-
	LR-F1 0.50 g/mL	-	-	-	-
	LR-F2 0.50 g/mL	-	-	-	-
	LR-F3 0.50 g/mL	-	-	-	-
Edema	NC	3	2	1	1
	PFD 0.08 g/mL	1	-	-	-
	Tween 80	2	1	-	-
	LR-F1 0.50 g/mL	2	1	-	-
	LR-F2 0.50 g/mL	2	-	-	-
	LR-F3 0.50 g/mL	-	-	-	-
Hemorrhage	NC	-	-	-	-
	PFD 0.08 g/mL	-	-	-	-
	Tween 80	-	-	-	-
	LR-F1 0.50 g/mL	-	-	-	-
	LR-F2 0.50 g/mL	-	-	-	-
	LR-F3 0.50 g/mL	-	-	-	-
Crust	NC	1	3	3	2
	PFD 0.08 g/mL	3	5	1	-
	Tween 80	1	3	3	2
	LR-F1 0.50 g/mL	2	5	-	-
	LR-F2 0.50 g/mL	2	5	-	-
	LR-F3 0.50 g/mL	2	5	-	-
Granulation	NC	-	-	-	1
	PFD 0.08 g/mL	-	-	2	5
	Tween 80	-	-	-	2
	LR-F1 0.50 g/mL	-	-	2	5
	LR-F2 0.50 g/mL	-	-	3	5
	LR-F3 0.50 g/mL	-	-	1	5

This table presents the clinical parameters observed in skin wounds following the topical application of the first fractionation groups of *B. bipinnata* resin (LR-F1, LR-F2, and LR-F3). The clinical parameters include wound closure percentages, signs of inflammation, edema, scab formation, and other relevant observations taken at multiple time points (Days 2, 7, 12, and 14). The treatments were compared with the negative control (NC). Each group had a sample size of $n = 5$.

Table S10. Clinical parameters observed in skin wounds after topical application of subfractions (LR-F2-A, LR-F2-B, LR-F2-C, LR-F2-D, and LR-F2-E) and treatments (Negative Control, PFD, and Vehicle).

**Parameters**	**Treatments**	**Days after wound induction**			
		**Day 2**	**Day 7**	**Day 12**	**Day 14**
Exudate	CN	2	-	-	-
	PFD 0.08 g/mL	2	-	-	-
	Tween 80	-	-	-	-
	LR-F2-A 0.50 g/mL	-	-	-	-
	LR-F2-B 0.50 g/mL	-	-	-	-
	LR-F2-C 0.50 g/mL	1	-	-	-
	LR-F2-D 0.50 g/mL	1	-	-	-
	LR-F2-E 0.50 g/mL	-	-	-	-
Edema	CN	3	2	1	1
	PFD 0.08 g/mL	1	-	-	-
	Tween 80	2	1	-	-
	LR-F2-A 0.50 g/mL	1	-	-	-
	LR-F2-B 0.50 g/mL	2	-	-	-
	LR-F2-C 0.50 g/mL	3	-	-	-
	LR-F2-D 0.50 g/mL	-	-	-	-
	LR-F2-E 0.50 g/mL	-	-	-	-
Hemorrhage	CN	-	-	-	-
	PFD 0.08 g/mL	-	-	-	-
	Tween 80	-	-	-	-
	LR-F2-A 0.50 g/mL	-	-	-	-
	LR-F2-B 0.50 g/mL	-	-	-	-
	LR-F2-C 0.50 g/mL	-	-	-	-
	LR-F2-D 0.50 g/mL	-	-	-	-
	LR-F2-E 0.50 g/mL	-	-	-	-
Crust	CN	1	3	3	2
	PFD 0.08 g/mL	3	5	1	-
	Tween 80	1	3	3	2
	LR-F2-A 0.50 g/mL	3	5	2	-
	LR-F2-B 0.50 g/mL	1	4	3	-
	LR-F2-C 0.50 g/mL	1	3	3	1
	LR-F2-D 0.50 g/mL	1	3	2	1
	LR-F2-E 0.50 g/mL	2	5	2	-
**Granulation**	CN	-	-	-	1
	PFD 0.08 g/mL	-	-	2	5
	Tween 80	-	-	-	2
	LR-F2-A 0.50 g/mL	-	-	3	5
	LR-F2-B 0.50 g/mL	-	-	2	3
	LR-F2-C 0.50 g/mL	-	-	1	4
	LR-F2-D 0.50 g/mL	-	-	1	3
	LR-F2-E 0.50 g/mL	-	-	3	5

This table presents the clinical parameters observed in skin wounds following the topical application of subfractions of *B. bipinnata* resin (LR-F2-A, LR-F2-B, LR-F2-C, LR-F2-D, and LR-F2-E). Clinical parameters include wound closure percentages, signs of inflammation, edema, scab formation, and other relevant observations at multiple time points (Days 2, 7, 12, and 14). The results were compared against the negative control (NC). Each group consisted of $n = 5$.

Table S11. Clinical parameters observed in skin wounds after topical application of the compounds: Stigmasterol (**1**), Lupenone (**3**), and 3-Epilupeol (**5**), and Treatments (Negative Control, PFD, and Vehicle).

Parameters	Treatments	Days after wound induction			
		Day 2	Day 7	Day 12	Day 14
Exudate	NC	2	-	-	-
	3-epilupeol 1 μM	1	-	-	-
	Stigmasterol 1 μM	-	-	-	-
	Lupenone 1 μM	-	-	-	-
Edema	NC	3	2	1	1
	3-epilupeol 1 μM	2	1	-	-
	Stigmasterol 1 μM	-	1	-	-
	Lupenone 1 μM	-	-	-	-
Hemorrhage	NC	-	-	-	-
	3-epilupeol 1 μM	-	-	-	-
	Stigmasterol 1 μM	-	-	-	-
	Lupenone 1 μM	-	-	-	-
Crust	NC	1	3	3	2
	3-epilupeol 1 μM	-	3	1	-
	Stigmasterol 1 μM	-	4	-	-
	Lupenone 1 μM	-	3	2	-
Granulation	NC	-	-	-	1
	3-epilupeol 1 μM	-	-	3	5
	Stigmasterol 1 μM	-	-	4	5
	Lupenone 1 μM	-	-	3	4

This table summarizes the clinical parameters observed in skin wounds following the topical application of isolated compounds from *B. bipinnata* resin: stigmasterol (**1**), lupenone (**3**), and 3-epilupeol (**5**). The parameters include wound closure percentages, signs of inflammation, edema, and scab formation at various time points (Days 2, 5, 7, 9, 12, and 14). The treatments were compared with the negative control (NC). Each group consisted of $n = 5$.
